# The redox aspects of lithium-ion batteries†

**DOI:** 10.1039/d4ee04560b

**Published:** 2024-12-14

**Authors:** Pekka Peljo, Claire Villevieille, Hubert H. Girault

**Affiliations:** a Research Group of Battery Materials and Technologies, Department of Mechanical and Materials Engineering, University of Turku FI-20014 Turun Yliopisto Finland pekka.peljo@utu.fi; b LEPMI, Univ. Grenoble Alpes, Univ. Savoie Mont Blanc, CNRS, Grenoble INP, LEPMI Grenoble France; c Institute of Chemical Science and Engineering, Station 6, Ecole Polytechnique Federale de Lausanne CH-1015 Lausanne Switzerland hubert.girault@epfl.ch; d Material Science and Nanoengineering (MSN) Department, University Mohammed VI Polytechnic 43 150 Ben Guerir Morocco; e Department of Chemistry and Materials Science, Aalto University P.O. Box 16100 Aalto Espoo 00076 Finland

## Abstract

This article aims to present the redox aspects of lithium-ion batteries both from a thermodynamic and from a conductivity viewpoint. We first recall the basic definitions of the electrochemical potential of the electron, and of the Fermi level for a redox couple in solutions. The Fermi level of redox solids such as metal oxide particles is then discussed, and a Nernst equation is derived for two ideal systems, namely an ideally homogenous phase where the oxidised and reduced metal ions are homogeneously distributed and two segregated phases where the oxidised and the reduced metal ions are separated in two distinct phases such as observed, for example, in biphasic lithium iron phosphate. The two different Nernst equations are then used to explain the difference in conductivity, the former being more conductive due to redox conductivity.

## Abstract 2

We are considering fundamental questions of how redox reactions take place and charge is conducted in redox solids. Redox properties of electrode materials can be explained by considering the Nernst equations for homogeneous or segregated materials.

## Graphical abstract

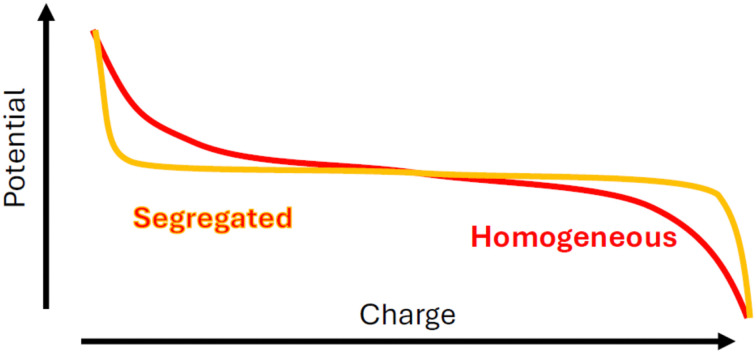

**Broader context**

Li-ion batteries have transformed the portable electronics and are crucial for green transition particularly for electric mobility, as recognized by the 2019 Nobel Prize in Chemistry awarded to John B. Goodenough, M. Stanley Whittingham and Akira Yoshino. The lithium battery research has mostly been carried out by materials scientists, with only moderate input from electrochemists. The field has been driven by progresses in solid state chemistry, and the electrochemical aspects were somehow overlooked or superficially addressed, and not easy to find for those just entering the field. In this perspective, we consider the fundamental questions of how redox reactions take place and charge is actually conducted in redox solids. Redox properties of electrode materials can be explained by considering the Nernst equations for homogeneous or segregated materials. Analogously to redox polymers, the conductivity of the materials is affected by the concept of redox conductivity.

## 1. Introduction

Over the last decades, the field of lithium batteries has evolved to be an integral part of any energy transition strategy, in particular for mobility applications.^1^ From a scientific viewpoint, the field has been driven by progresses in solid state chemistry, and the electrochemical aspects were somehow overlooked or superficially addressed, with focus mostly on methodology.^2^ The purpose of this article is first to recall some fundamental concepts for redox electrochemistry in electrolyte solutions and address a well-spread misconception regarding the graphical representation of the Fermi level of the electron in solution. The second goal of this paper is to address the electrochemistry of redox solids, either in a dry state or in solution, and present simple expressions of the Nernst equation for two ideal systems. We shall differentiate homogeneous redox solids, so-called solid-solution reaction, and segregated redox solids, so called biphasic reaction, and show the differences not only in terms of the Nernst equations but also in terms of conductivities. Indeed, redox conductivity occurs in homogeneous systems whereas segregated systems are less conductive.

The Fermi level of a solid, *e.g.* metal, semi-conductor, insulator… is a key concept in solid state physics. It can be defined in different ways as:^3^

– The highest energy level that an electron can occupy at the absolute zero temperature (highest occupied molecular orbital, HOMO at 0° K).

– The thermodynamic work required to add one electron from vacuum.

– In the framework of the Fermi–Dirac statistics, it is defined as a level with a probability of occupation of ½.

Electrochemical potential of an electron in any system, *μ̃*_e^−^_, is defined as the thermodynamic work required to add one electron from vacuum to the system. Therefore, the Fermi level of any system is equivalent of the electrochemical potential of an electron in the given system.^4–6^ As discussed in Section 3 below, the electrochemical potential of an electron in any redox system can be expressed by the Nernst potential of the system. For large neutral phases, all the definitions are equivalent either from the point of zero energy as in solid state physics or from the electron at rest, in vacuum, as in electrochemistry for defining the electrochemical potential. It should be remembered that all phases are neutral inside and only charged on their surfaces. A neutral phase has no excess charges on its surface.

The concept of Fermi level is indeed a key concept in electrochemistry, when considering electron transfer between different phases, being either solids or liquids containing redox species or particles.^4–6^ Indeed, at equilibrium the electrochemical potential of electrons should be the same for the different phases. However, this concept should be handled with care in electrochemical charged systems.

## 2. Contact potential and the Fermi level of electronic conductors

When considering two electronic conductor phases in contact, electrons will flow from the metal with the higher Fermi level to the other, resulting in the two phases becoming charged, and the establishment of a potential difference between the two metals, called the outer potential difference or the Volta potential difference. The positive charges or the negative charges are located at the surface of each metal, and not only at the physical zone of contact.^7,8^

If the concept of contact potentials is very old and at the origin of electricity generation, it is worth discussing the graphical representation of Fermi levels when two metallic objects are placed in contact as some confusion can be observed in the literature.

**Fig. 1:**
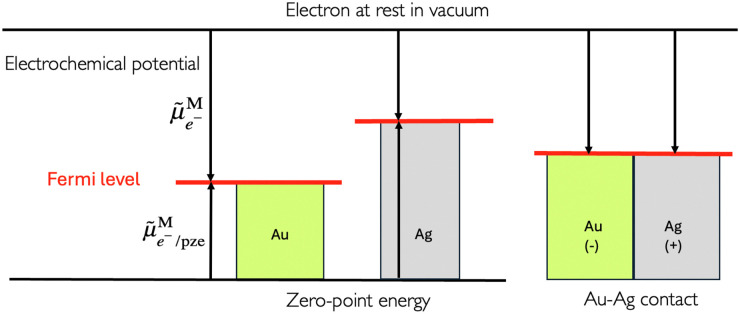
Electrochemical potential and Fermi levels for gold, silver and a silver–gold contact defined with respect to the electron at rest in vacuum for the separated and in contact metals.

In the scheme shown in Fig. 1, the Fermi level in an electronic conductor (EC) is defined from the zero-point energy and with respect to the electron at rest in vacuum. The electrochemical potential of the electron, *μ̃*_e^−^_, with respect to vacuum is defined as the sum of a “chemical” term including all the short-range interactions, *μ*_e^−^_, and an “electrostatic” term, −*Fϕ*, depending on the inner potential, also called Galvani potential, *ϕ*, which is itself the sum of the surface potential, *χ*, depending on the presence of a surface dipole, *e.g.* the Jellium model, and an outer potential, *ψ*, depending on the excess charge located at the surface of the metal. The basic concepts have been reviewed in detail from a point of view of an electrochemist, for example in ref. 7.1
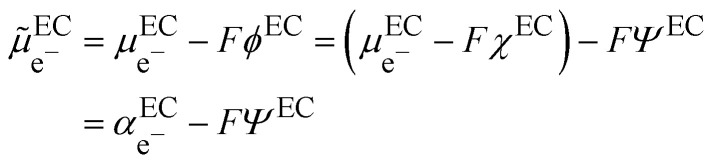
Here, 
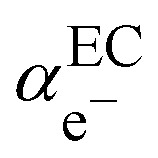
 represents the so-called “real” chemical potential, the charge independent part of the electrochemical potential equal to the opposite of the work function, Φ^EC^, defined as the work to extract an electron from an uncharged metal or any electronic conductor EC.2
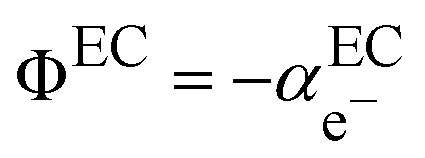
The real chemical potential depends on the crystallographic faces for single crystals but also on the presence of surface defects, to reflect the variation of the dipolar surface potential. When two electronic conductors EC^1^ and EC^2^ having different work functions are placed in contact, electrons will flow from the conductor having the smaller work function to the other and the difference of outer potentials will be directly related to the difference in work functions:

3 *ψ*^EC^2^^ − *ψ*^EC^1^^ = −(Φ^EC^2^^ − Φ^EC^1^^)/*F*

In a thermodynamic diagram, we have then an equality of the Fermi levels of the charged conductors. Nonetheless, it is important to remember that the charges are located at the surface of the conductor but that the bulk conductors remain neutral.

From a thermodynamic viewpoint at equilibrium, the electrochemical potential of the electron is equal for the two conductors defining a unique Fermi level of the electron for the two conductors in contact.4
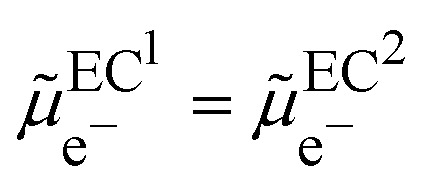
Contact potentials of charged metals have already been reviewed^7^ with emphasis on nanomaterials. Indeed, the variation of the Fermi level of a metal with the charge becomes more important with nanomaterials since the surface contribution plays a more important role with respect to that of the volume.^4^ This has major consequences, for example, when using nanoparticles for electrocatalysis and/or batteries.

Regarding lithium-ion batteries, carbon black or carbon coating is often used as an electron conductor. The Fermi level of the electron on the carbon varies to follow that of the contact electrode by changing its electronic interface charge and hence its outer potential.

## 3. Fermi level for a redox couple in solution

Before to address the redox aspects of lithium-ion batteries, it is worth recalling the fundamental basis of redox electrochemistry in solutions, as many misconceptions are present in the literature. This discussion is based on the textbook by Girault.^6^

### 3.1. The electrochemical potential of the electron in solution containing a redox couple

In an electrolyte solution containing a redox species ox/red, *e.g.* Fe^III^/Fe^II^ in water, we can define the electrochemical potential of the electron as the work to bring one electron from vacuum to the solution on the most reducible oxidized species (*e.g.* Fe^III^) that becomes reduced.

**Fig. 2:**
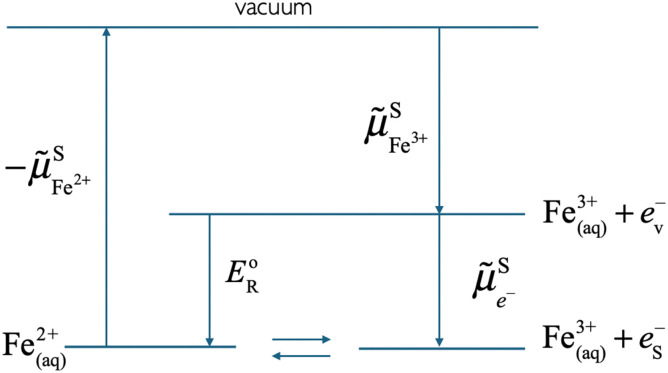
Thermodynamic cycle showing the electrochemical potential of the oxidised and reduced species, and the electrochemical potential of the electron in solution equal to the reduction energy between two optimally solvated redox species.

As shown in Fig. 2, the work to bring an electron is linked to the work to remove the reduced species 
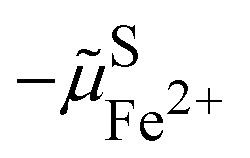
 and to add an oxidised species 
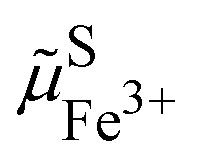
 and then to add the electron from vacuum, 
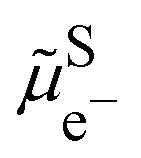
, to the oxidised species.

As can also be seen in Fig. 2, the electrochemical potential of the electron in solution corresponds to the reduction energy of an optimally solvated oxidised species to an optimally solvated reduced species.

### 3.2. Standard redox potentials

The standard redox potential of a redox reaction in a solution, S, on the absolute vacuum scale considers the following reaction.

I ox^S^ + e^−v^ ⇆ red^S^

It is defined from the electric work, Δ*G̃*^o^_r_, that can be recovered from this reaction in the standard conditions, *e.g.* on the molarity scale.5
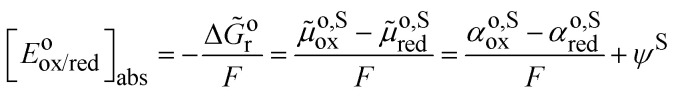
the last term being equal to zero for uncharged solutions.

The standard redox potential on the standard hydrogen electrode (SHE)^9^ is defined as the electric work that can be recovered from a cell containing an inert metal electrode in the redox solution and a platinum electrode immersed in an acid solution (pH = 0) in the presence of hydrogen gas at 1 bar separated by a diaphragm. The overall reaction readsII
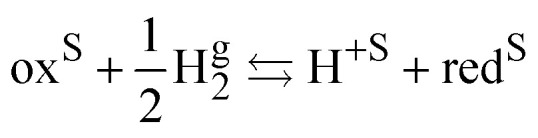
So, for a redox couple in solution able to exchange electrons with an inert electrode, the Nernst equation on the SHE scale is classically given by:6
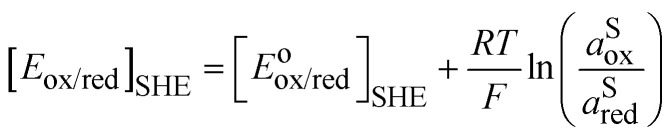
The challenge of eqn (6) is that activity coefficients of oxidized and reduced species are difficult to evaluate especially for concentrated electrolytes. The two scales are related by7
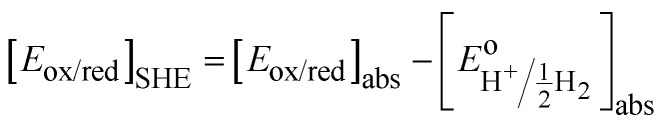
with8
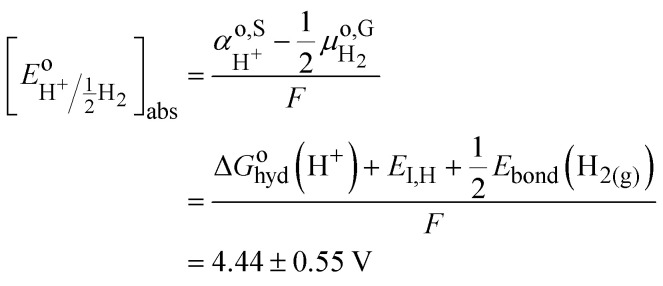
The two redox scales cannot be exactly matched, due to the difficulty of measuring exactly the hydration energy of the proton.^10,11^

### 3.3. Fermi–Dirac statistics for a redox reaction in solution

Following the Gerischer model of electron transfer reactions,^12^ a redox reaction in solution obeys to a two-state Fermi–Dirac statistics of the reduction energies, with the solvation energies of the oxidised and reduced species fluctuating with the solvent dynamics. This vocabulary is indeed a potential source of confusion as the definition of state here does not refer to the energy level of the electron on an oxidised or reduced species, but to reduction energies.

**Fig. 3:**
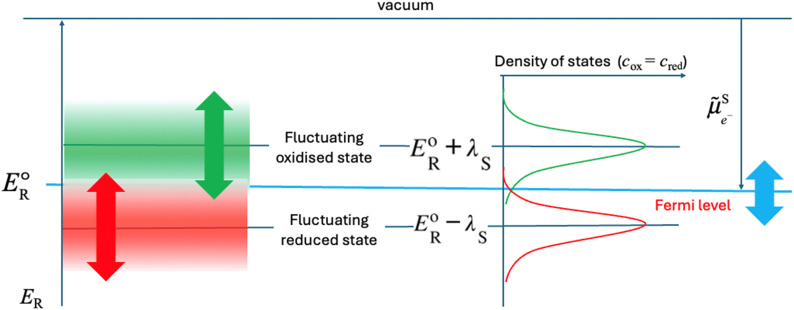
Two-state Fermi–Dirac distribution of the reduction energies for a redox reaction in solution.

When plotting the reduction energies as a function of the density of states, these two reduction energy states are separated by twice the solvent re-organisation energy, *λ*_S_ (see ESI†). In Fig. 3, the reduction energy *E*^o^_R_ corresponds to an electron transfer reaction where both the oxidised and reduced species are optimally solvated, as in Fig. 2. One state of this Fermi–Dirac statistics, called here oxidised state, corresponds to the case where the oxidised species is optimally solvated, and the associated reduced species is therefore very poorly solvated. In the case of Fe^III^/Fe^II^, Fe^III^ is optimally hydrated and the resulting Fe^II^ is a 2+ cation with a hydration shell of a 3+ cation, the electron transfer being much faster than the solvent relaxation. The energy of this state is *E*^o^_R_ + *λ*_S_. The other state of this Fermi–Dirac statistics, called here reduced state, corresponds to the case where the reduced species is optimally solvated and the oxidised one very poorly solvated. The energy of this state *E*^o^_R_ − *λ*_S_. As illustrated in Fig. 3, the reduction energies are by definition negative as electronegativity, and if *E*_R_ − *E*^o^_R_ < 0, the reduction is easy and the ox/red couple is mainly in the reduced form, whereas if *E*_R_ − *E*^o^_R_ > 0, then the reduction is difficult and the ox/red couple is mainly in the oxidised form.

In Fig. 3, the Gaussian distribution of the density of states stems from the assumption that the solvent polarisation is harmonic (see ESI†). It is important to recall that these Gaussians DO NOT represent an energy band containing many energy levels, but the fluctuation with the solvent polarisation of a single state. The height of the Gaussian is proportional to the concentration of ox and red, which are linked by the relation:

9 *c*_ox_ + *c*_red_ = *c*_tot_

**Fig. 4:**
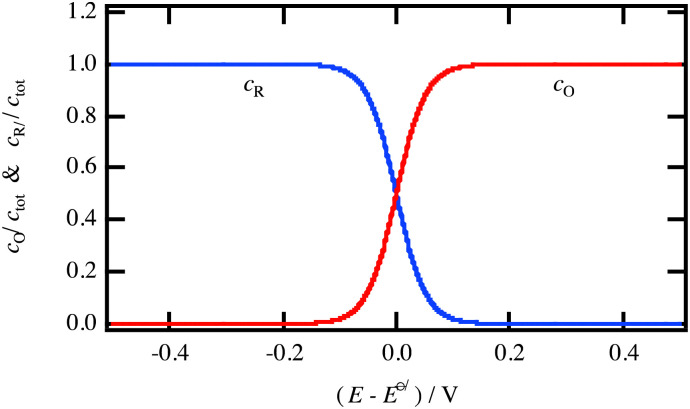
Concentration dependence of the reduced and oxidised species for a redox reaction in solution as a function of the electrode potential.

The intersection of the two Gaussian curves defines the Fermi level of this two-state statistics, as the probability of occupation is equal to 1/2. The Fermi level, *E*_F_R__, varies then with the ratio *c*_ox_/*c*_red_:10
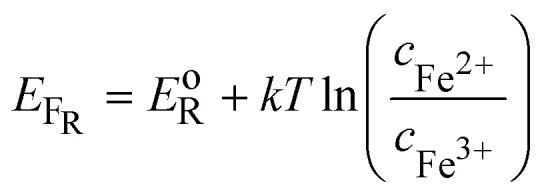
A direct comparison with the Nernst eqn (6) shows that:

11 *E*_F_R__ − *E*^o^_R_ = −*F*[*E*_ox/red_ − *E*^o/^_ox/red_]_SHE_

where *E*^o/^_ox/red_ is the formal potential being the sum of the standard redox potential and an activity coefficient term *RT* ln(*γ*_ox_/*γ*_red_)/*F*. The concentration profiles as a function of the potential are then:12
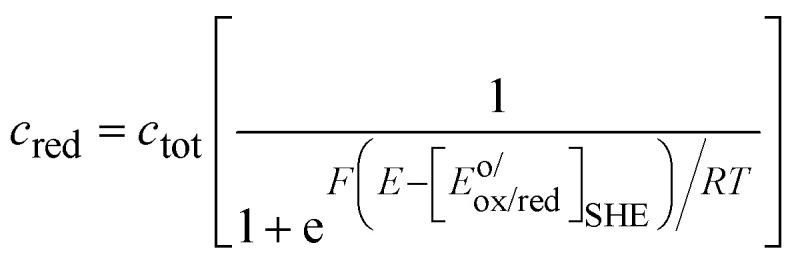
and13
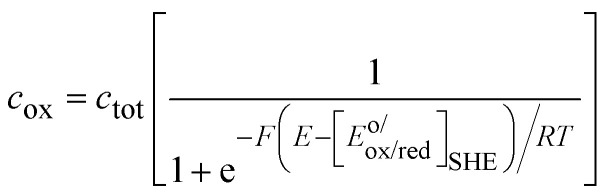
as illustrated in Fig. 4, and characteristic of a two-state Fermi–Dirac statistics.

**Fig. 5:**
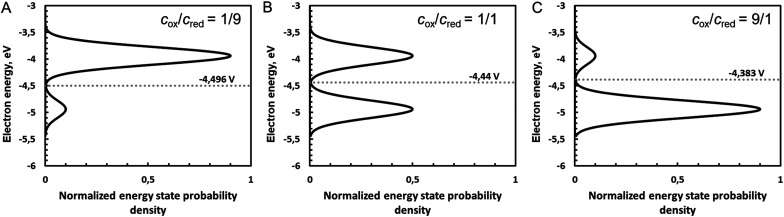
Influence of the ratio *c*_ox_/*c*_red_ on the density of states and hence of the Fermi level defined as the intersection of the two Gaussian distributions for *c*_ox_/*c*_red_ ratios of (A) 1/9, (B) 1/1 and (C) 9/1. Gaussian distributions are calculated with eqn (A38) and (A39) (ESI†), with *E*^o^ taken as 4.44 V on the absolute vacuum scale (AVS), with standard hydrogen electrode assumed to be at 4.44 V. Conversion to electron energy scale is done by multiplying the potential of the AVS scaly by charge of an electron −e. Note that potential increases down, *i.e.* Gaussian distribution of the oxidized species is below. Here, *λ* was taken as 0.5 eV.

Fig. 5 shows the influence of the ratio *c*_ox_/*c*_red_ on the density of states and hence of the Fermi level defined as the intersection of the two Gaussian distribution, calculated with eqn (A38) and (A39) in the ESI.†

### 3.4. Fermi level for the electron in solution

**Fig. 6:**
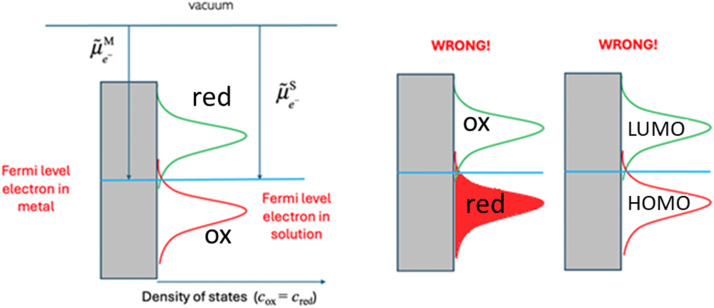
Comparison of the Fermi levels for a redox reaction on an electrode.

From the above discussion, we see that the electrochemical potential of the electron in solution is linked to the Fermi level of the electron in solution and corresponds to the Nernst redox potential on the absolute vacuum scale:14
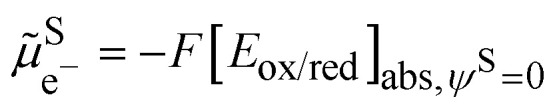
Eqn (14) is widely used in electrochemistry for electrode reactions to compare to the Fermi level of the electron on a metal and in solution as shown in Fig. 6. When the former is lower than the latter, we have an oxidation of the species in solution, and when the former is higher than the latter, we have a reduction of the species in solution.

A major misconception when describing Fig. 6 is to ascribe the two Gaussian to hypothetic energy level of the electron in solution, where the bottom Gaussian is attributed to a filled reduced state (electron present on the reduced species) and the upper Gaussian to an empty oxidised state (vacant electron energy level). In fact, the bottom Gaussian describes the density of states of the oxidized species, while the top Gaussian is for the reduced species. A further misconception is to call the bottom Gaussian curve a HOMO energy level and the upper Gaussian a LUMO energy level.

The correct way to discuss Fig. 6 is to compare the electrochemical potential of the electron on the metal and in solution. As we have shown in our earlier work, HOMO and LUMO are concepts derived from approximated electronic structure theory while investigating electronic properties of isolated molecules, and their energy levels do not indicate species participating in redox reactions. On the other hand, redox potentials are directly related to the Gibbs energy difference of the reactants and products as defined by eqn (5).

## 4. The Fermi level for a redox solid in solution

### 4.1. The electrochemical potential of the electron in solution containing redox solid particles

When dealing with a redox solid particle such as a metal oxide with inserted cations, *e.g.* LiMO_2_ with M = Mn, Ni or Co, the Fermi level is often calculated *ab initio*, using for example DFT calculations, for the reduced and oxidized neutral solid separately. The variation of the density of states as a function of the energy is then referred to the zero-point.

From an electrochemical standpoint, a redox solid LiMO_2_ is similar to a redox solution, and the Fermi level can be defined by the redox potential of the redox couple (*e.g.* M^IV^/M^III^). If the oxide remains an ideally homogeneous phase upon charge transfer reaction, *i.e.* if there is no phase segregation between the oxidised and reduced species, the Fermi level of the redox solid will vary with the ratio *c*_ox_/*c*_red_.

**Fig. 7:**
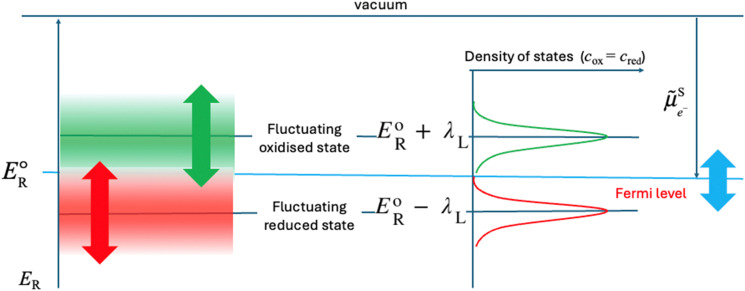
Two-state Fermi–Dirac distribution of the reduction energy for a redox reaction in a solid assuming a harmonic variation of the re-organisation energy.

The major difference between liquid and solid redox phases is the solvent re-organisation, *λ*_S_, for the former and the lattice re-organisation energy, *λ*_L_, in the presence of the inserted cations for the latter. This has been discussed in detail for example in ref. 13–16. As discussed above, the fluctuations of the polarisation of the solvent affect the solvation of the ions and hence their reduction energy. In a solid, the re-organisation of the lattice and the motion of the inserted cations will generate defined states of the reduction energy as illustrated in Fig. 7.

In solids, the oxidised state corresponds to the case where the oxidised species is optimally coordinated in the lattice and the associated reduced species very poorly coordinated. In the case of M^IV^/M^III^, M^IV^ is optimally coordinated with the oxygen cage and the resulting M^III^ is a 3+ cation with an environment of a 4+ cation in the absence of a lithium ion, the electron transfer being much faster than the lattice relaxation and the motion of the inserted cation. The energy of this state is *E*^o^_R_ + *λ*_L_. The reduced state corresponds to the case where the reduced species is optimally coordinated together with the inserted cation and the oxidised one very poorly coordinated. The energy of this state is *E*^o^_R_ − *λ*_L_. The density of states is more complicated in solids, as there is no solvent to reorganize, but the redox reactions are coupled with intercalation.

For redox reactions in solution, the presence of an excess of supporting electrolyte means that we do not consider the need to maintain electroneutrality when writing the redox reaction. This is of course not the case in a redox solid where the electrochemical reaction is balanced by ion insertion and extrusion to maintain the core of the solid neutral. For example,

III CO^IV^O_2_ + Li^+S^ + e^−^ ⇄ Li^+^Co^III^O_2_

where Li^+S^ refers to a lithium ion in a non-redox electrolyte solution. The lithiated oxide can ideally be considered as an ionic solid Li^+^ [Co^III^O_2_]^−^, where the cation is mobile.

### 4.2. Changing the Fermi level of a redox solid without cation insertion–extrusion

**Fig. 8:**
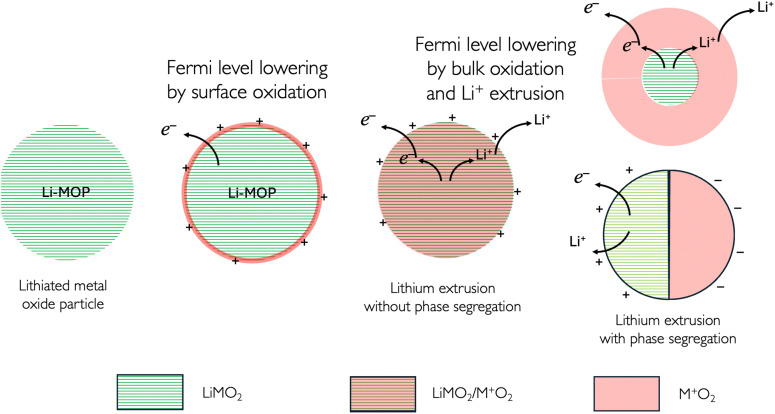
Redox reactions on and in a lithiated metal oxide particle (MOP). The segregated phases are illustrated here in a core–shell manner (top), or segregated manner (bottom) in a contact mode, *e.g.* two hemispheres.

To change the Fermi level of the redox solid, we can first vary the outer potential by charging the surface as illustrated in Fig. 8. In the case of a lithiated metal oxide, the oxidation of the surface metal cations will lead to a positively charged particle. This surface charge will be compensated by an anion from the electrolyte solution, forming a Helmholtz layer of anions, *e.g.* PF_6_^−^, on the charged metal oxide particles (MOP).^17^

The presence of a positive charge on the particle will decrease (more negative) the electrochemical potential of the electron on the particle and will lower the Fermi level as the work function increases.

Upon further oxidation, the transition metal species of the bulk material becomes oxidised either in a homogeneous or in a segregated manner as shown in Fig. 8, and this reaction is coupled with deintercalation of the Li^+^.

### 4.3. Nernst equation for lithium insertion in an ideally homogeneous metal oxide particle

**Fig. 9:**
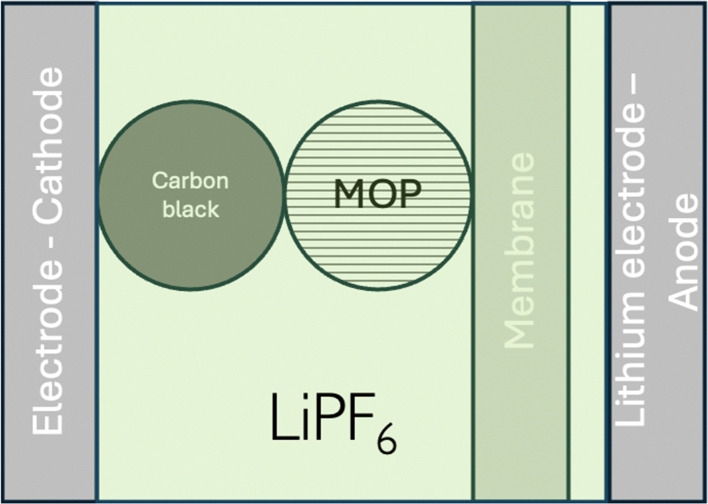
Schematic illustration of a LiMO_2_ lithium battery.

Let us consider, LiMO_2_, a so-called, positive electrode material for lithium-ion batteries as ideally homogeneous redox solid. From a simplified electrochemical standpoint, such a lithium-ion battery can be illustrated as shown in Fig. 9. From a semantic viewpoint, the positive electrode during discharge in Fig. 9 is the aluminium current collector (Al) and the carbon particle whereas the negative electrode during discharge is the lithium metal on the current collector (Cu). It should be stressed that LiMO_2_ is a redox particle in solution and not an electrode as often written in the literature that is why it is better to refer to either redox particle or to electroactive materials rather than electrode materials.

If we assumed that the positive charges within the metal oxide particles are localized on the metal ions and lithium cations, the electrochemical reaction during charge is

IV M^IV^O_2_ + Li^+S^ + e^−CB^ ⇄ Li^+^M^III^O_2_

the electron being provided by the conductive agent, *i.e.* carbon black (CB) particle. It is important to emphasize that the reaction is a triphasic reaction. At equilibrium, we have the equality of the electrochemical potentials of the products and the reactants of reaction (IV):15
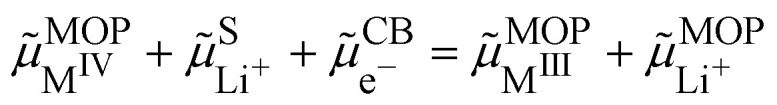
where CB stands for carbon black acting as an electrode, MOP for the metal oxide redox particle and S the electrolyte solution. By developing the electrochemical potentials with a standard term, an activity term and an electric term, we have16
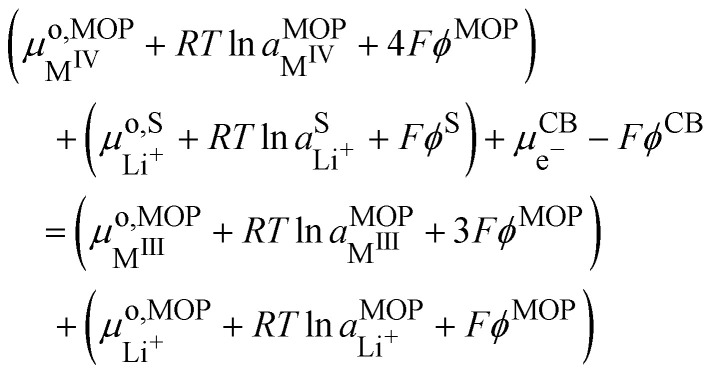
We can then calculate the Galvani potential difference between the carbon electrode and the electrolyte solution in which the redox LiMO_2_ particles are bathing17
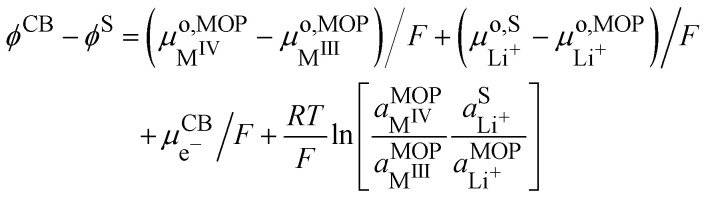
A lithium metal electrode can be used as a negative electrode to refer the Nernst potential to a lithium metal reference.

V Li^+S^ + e^−Li^ ⇆ Li^metal^

The Nernst equation for reaction (V) is simply:18
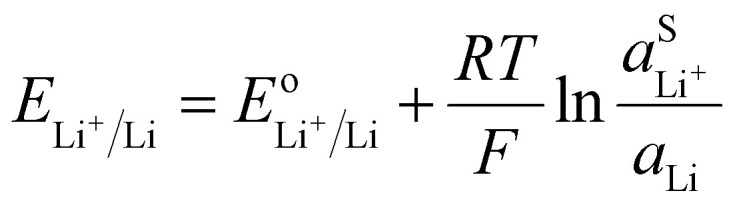
The activity of Li metal can be considered as 1 if the metal is pure. Therefore, the Nernst potential to a lithium metal electrode depends only of the activity of Li^+^ in the electrolyte. For utilization of the lithium metal reference, the activity of the Li^+^ in the electrolyte should be unity resulting in the standard potential. However, a standard state should be defined for example on the molarity scale, such that a one molar lithium electrolyte solution has a lithium ion activity of 1 when neglecting the activity coefficients. Eqn (18) can be written in general form for any soluble/insoluble system, for example for Zn/Zn^2+^, Cu/Cu^2+^, Na/Na^+^, *etc.*

In this case, the cell voltage defined as the Galvani potential difference between the two metallic contacts (MC^I^ and MC^II^) connected to the positive and negative electrode respectively is given by:19
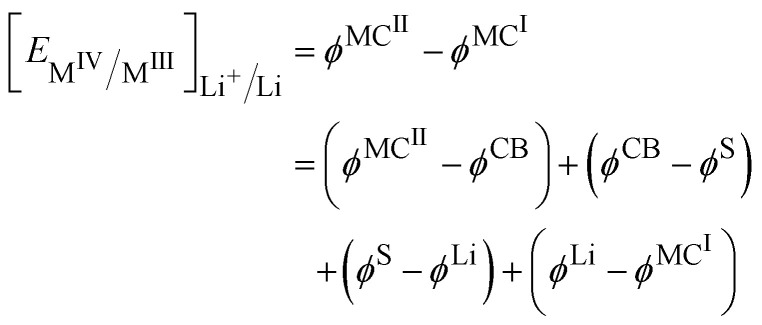
Considering the equality of the electrochemical potential of the electron in M^II^ and the carbon black, we have20
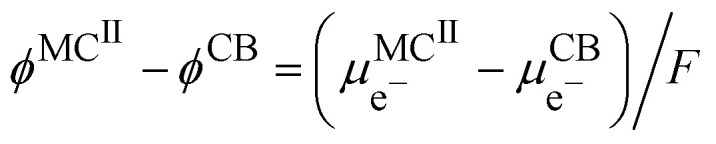
At the lithium counter/reference electrode, the Galvani potential difference between the lithium metal and the electrolyte solution is given by21
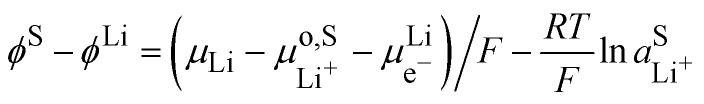
By using the equality of the electrochemical potential of the electron in the lithium metal and M^I^, we can write22
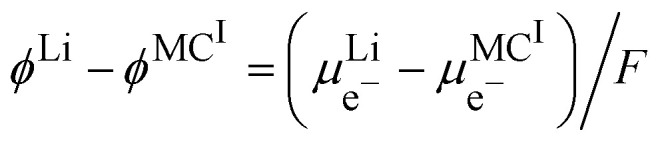
Finally, eqn (18) reduces to:23
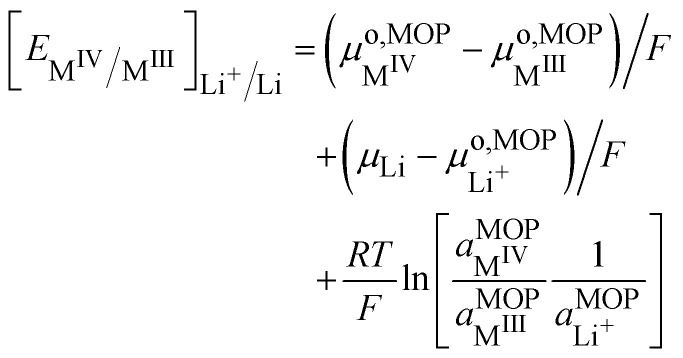
Eqn (23) is the Nernst equation for an ideally homogeneous LiMO_2_/MO_2_ redox solid, showing that the Fermi level varies with the ratio of the activities of the different ionic species involved in the redox reaction.

**Fig. 10:**
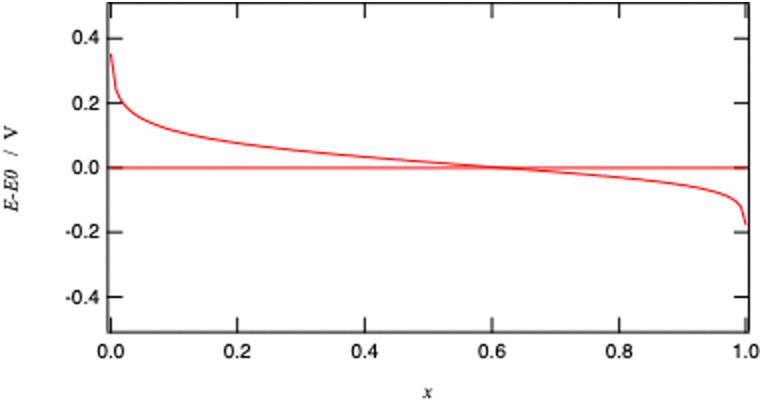
Nernst potential for an ideal homogeneous metal oxide particle from fully oxidised (*x* = 0) to fully reduced (*x* = 1) form.

By regrouping the standard terms, we can define the standard redox potential, 
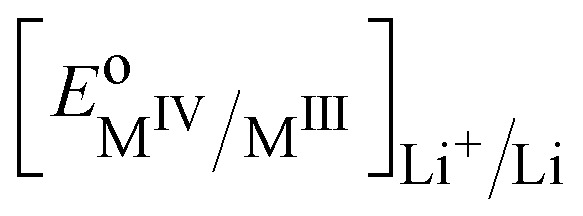
 for the redox couple M^IV^/M^III^ with respect to the lithium reference electrode in the solvent used for dissolving the lithium electrolyte24
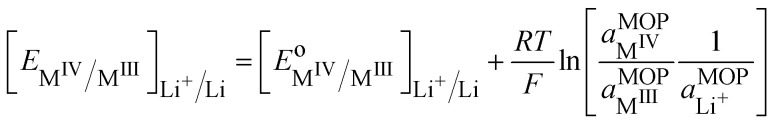
Eqn (24) is a general case, but the challenge in practise is to evaluate the activities of different species. We can express the activities in terms of molar fractions with respect to a total concentration of metal atoms, which by neglecting the activity coefficients gives:25
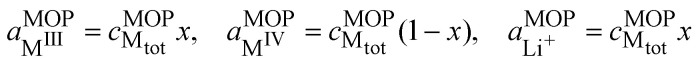
The Nernst equation of the ideally homogeneous positive electrode then simply reads26
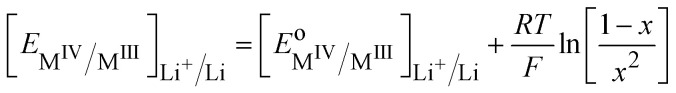
Here, the *x*^2^ term stems from the fact that we have considered Li^+^ and M^III^ in the redox solid as independent ionic quantities. Similar expression with *x* instead of *x*^2^ is obtained by considering Li^+^M^III^ as a single species participating in the reaction, which is erroneous. Fig. 10 illustrates the Nernst potential for an ideally homogeneous LiMO_2_ positive electrode material. The standard redox potential corresponds to *x* = 0.618 if we neglect the activity coefficient.

Equivalent of eqn (26) can be derived for any intercalation material where oxidation/reduction within the solid is coupled with intercalation. As an end result, M^IV^ an M^III^ are replaced with the redox species in question, for example with Fe^III^ and Fe^II^ in Prussian blue analogues or –C–O^−^/–C=O in organic batteries, and Li^+^ is replaced by the intercalating cation. Similar equations can be derived for anion intercalation. More complex materials such as LiMn_2_O_4_ containing different redox sites at different potentials require treatment of each redox site separately, with Nernst equations for each different redox site. This system could be considered analogously to a mixture of multiple redox couples in solution. This kind of treatment would also be required for example for different soluble polysulfide species.

Considering that *x* is often assimilated to the charge of the battery, Fig. 10 is therefore analogous to potential *versus* charge plot, often presented to characterise a battery performance. Measuring the cell voltage is therefore a means to follow the advancement of the charging process.

It is important to stress that the Nernst eqn (26) is valid for a homogenous redox solid where the two metallic redox ions M^IV^ and M^III^ are homogeneously distributed as if they were individual ions in an electrolyte solution.

The Nernst eqn (24) or (26) provides the electrical work that can be recovered from the virtual reaction between the oxidised metal oxide and lithium metal to form the lithiated and reduced metal oxide.

VI M^IV^O_2_ + Li ⇄ Li^+^M^III^O_2_

**Fig. 11:**
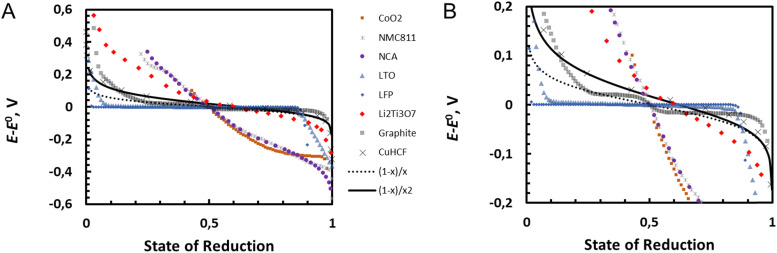
Comparison of experimental potential curves for eqn (26) with ln[(1 − *x*)/*x*^2^] and corresponding curve ln[(1 − *x*)/*x*] where reduced redox solid is considered as single quantity. The zoom in on the potential range −0.2 to 0.2 V is shown on the right.

Fig. 11 shows experimental potentials for different lithium intercalation electrode materials cobalt oxide (CoO_2_), nickel manganese cobalt oxide (NMC811, Ni_0.8_Mn_0.1_Co_0.1_O_2_), nickel cobalt aluminium oxide (NCA, Ni_0.84_Co_0.12_Al_0.04_O_2_), lithium titanium oxide (LTO, TiO_2_), lithium iron phosphate (LFP, FePO_4_) and graphite taken from COMSOL database^18^ and for lithium titanate Li_2_Ti_3_O_7_ from ref. 19 and potassium intercalation material copper hexacyanoferrate (CuHCF) from ref. 20 as the function of state of reduction (for fully reduced material this is 1). The standard potential is estimated as some value close to state of reduction of 0.5 for comparison of the curves.

Oxide materials CoO_2_, NMC811, NCA show very steep change of potential (super-Nernstian behaviour), Li_2_Ti_3_O_7_ more moderate super Nernstian behaviour while LFP and LTO show very flat plateaus (sub-Nernstian behaviour). Graphite shows several different sub-Nernstian plateaus, and only CuHCF shows agreement with eqn (26). Eqn (26) represents the Nernst equation for an ideal homogeneous MO_2_/LiMO_2_ redox solid, where the activity coefficients are not considered, and no phase transitions are assumed to occur. The non-idealities including phase transitions can be considered by fitting the activity coefficients for example using the Redlich–Kister model,^21^ or by thermodynamic description of the phase transitions in so-called CALPHAD approach (CALculation of PHAse Diagrams).^22^ In CAPLPHAD, a set of thermodynamic descriptions of Gibbs energy of each single phase is established with specific models with adjustable parameters.^22^ Alternatively, thermodynamic parameters for phase diagrams as well as for state-of-charge curves can be obtained from quantum chemical calculations as described in some recent reviews.^23,24^

### 4.4. Lithium ion insertion and Galvani potential difference

It is interesting to see that the Li^+^ insertion/extrusion reactions between the solution and the metal oxide particle (MOP)

VII Li^+,S^ ⇄ Li^+,MOP^

are driven by the Galvani potential difference between the MOP and the electrolyte solution. Indeed, in terms of electrochemical potentials, the equilibrium (VII) yields:27
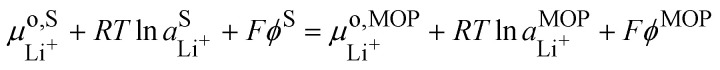
The standard state for lithium in the metal oxide particle corresponds to the fully lithiated phase, from which we obtain what is often referred to as the Nernst equation for an ion transfer reaction28
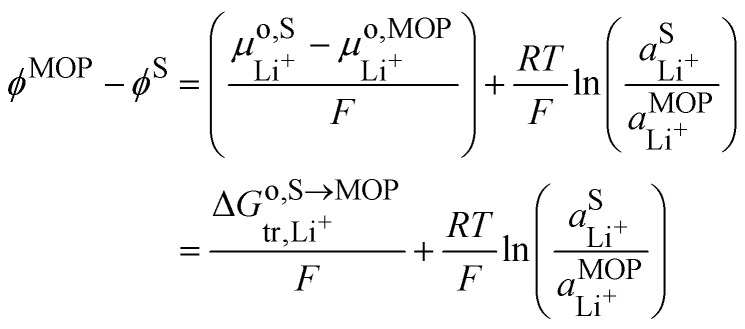
where 
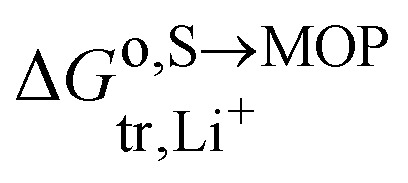
 is the standard transfer energy of lithium cation from the solution to the metal oxide particle. Eqn (28) expresses the Galvani potential difference between the MOP and the electrolyte solution as a function of the lithiation, whereas eqn (17) expresses the Galvani potential difference between the carbon black particle, *i.e.* the electrode, and the solution.

By combining eqn (17) and (28), we obtain the Galvani potential difference between the carbon black electrode and the metal oxide particle.29
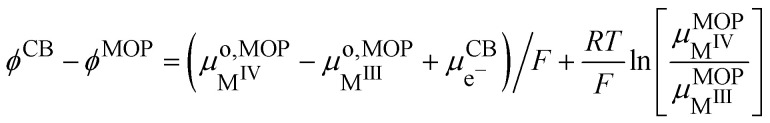
This equation shows that the Galvani potential difference is independent of the lithium ion concentration either in the solid or in the electrolyte solution. This is somehow a Nernst equation that shows Galvani potential difference between the carbon black electrode and the redox particle depends only on the ratio M^IV^/M^III^.

### 4.5. Contact potential between segregated phases

**Fig. 12:**
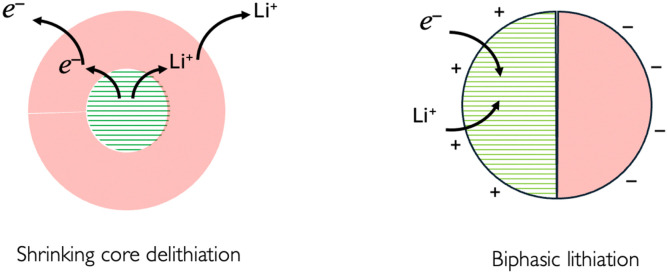
Schematic of core de-lithiation and biphasic lithiation as observed from microscopy experiments, as described in ref. 25.

If upon charging and due phase change the MOP becomes in parts fully delithiated and in parts fully lithiated as can be schematically seen in Fig. 12, the triphasic reaction (III) becomes quadriphasic: a lithium rich MOP, a lithium poor MOP, a carbon black electron donor/acceptor and the electrolyte solution but overall, the reaction can still be written

VIII M^IV^O_2_ + Li^+S^ + e^−CB^ ⇆ Li^+^M^III^O_2_

As an example in the case of LCO electroactive material, it has been shown^25^ by using optical interferometric scattering microscopy that delithiation occurs preferentially by a shrinking core mechanism and that lithiation occurs through a biphasic transition. This difference was explained in terms of the differences in lithium diffusivity in the two phases, with support from phase field modelling.

The “shrinking core” mechanism during lithium extrusion is attributed to a higher lithium flux across the active electrochemical surface to the electrolyte, as compared to the lithium flux inside the particle. In contrast, the intercalation mechanism was called ‘charge-transfer-limited’ resulting in the formation of a phase front propagating across the particle as shown in Fig. 12, between with a semiconducting lithium-rich phase, of approximate composition Li_0.95_CoO_2_, to the metallic lithium-poor phase, of approximate composition Li_0.77_CoO_2_.

The two phases being in contact the electrochemical potential of the electron and hence Fermi level of the electron is the same for the two phases if the equilibrium is reached. Nonetheless, there is a contact potential difference. Indeed, if we have phase segregation, we introduce a contact potential difference between the two metal oxide phases. By considering the electrochemical potential of the electron in ideally pure M^IV^O_2_ in contact with an ideally pure phase of Li^+^M^III^O_2_, as for two metals in contact, there must be an electronic equilibrium,30
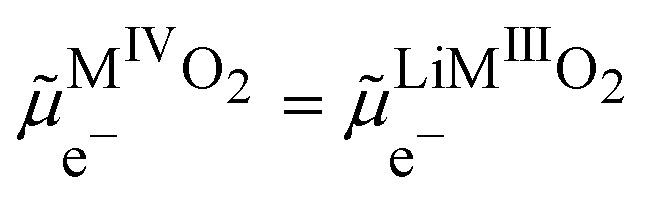
and we have a difference of outer potentials reflecting the difference of work functions.31
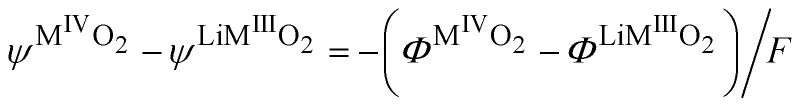
M^IV^O_2_ will be positively charged and Li^+^M^III^O_2_ negatively charged, and hence the counter ions adsorption from the electrolyte solution will differ.

Eqn (30) can also be used to express the difference of Galvani potential between the two oxide phases, the fully lithiated one and non lithiated one.32
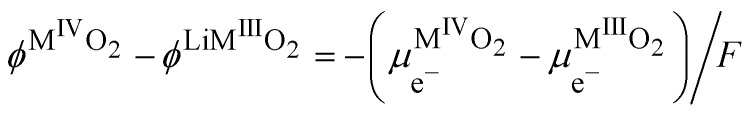


### 4.6. Nernst equation for lithium insertion in segregated metal oxide particles

We can now consider ideally segregated Li^+^M^III^O_2_ and M^IV^O_2_ immersed in a lithium electrolyte. Overall, we can write the quadriphasic reaction

VIII M^IV^O_2_ + Li^+S^ + e^−CB^ ⇄ Li^+^M^III^O_2_

but now we must distinguish two solid phases namely, pure M^IV^O_2_ referred to as MOPIV and pure lithiated Li^+^M^III^O_2_ referred to as MOPIII. The equality of the electrochemical potential for reaction (VIII) reads33
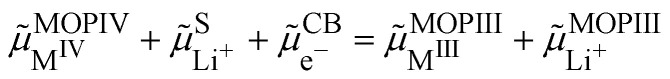
or by developing34
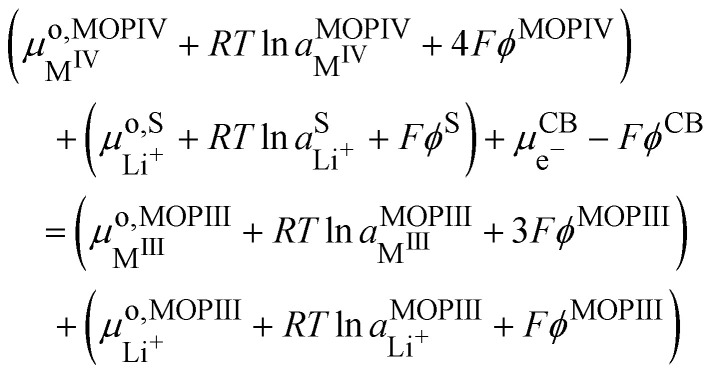
Still considering lithium metal as negative counter/reference electrode, we have35
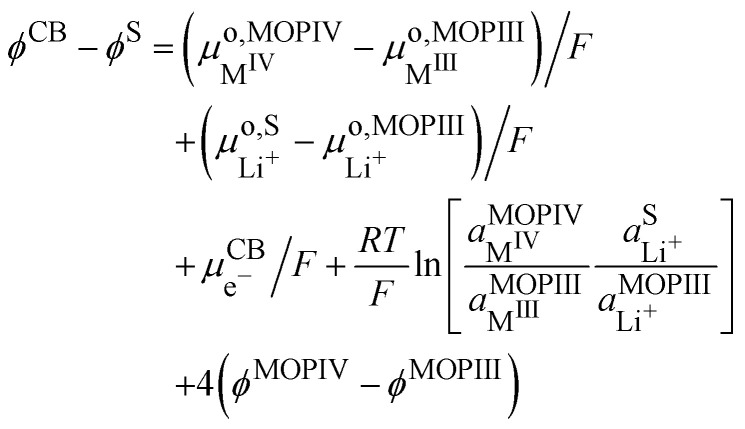
By introducing eqn (31), and by considering that the activity of the pure phases are unity we obtain the Nernst equation that shows that in this ideal segregated case, the Nernst potential is constant.36
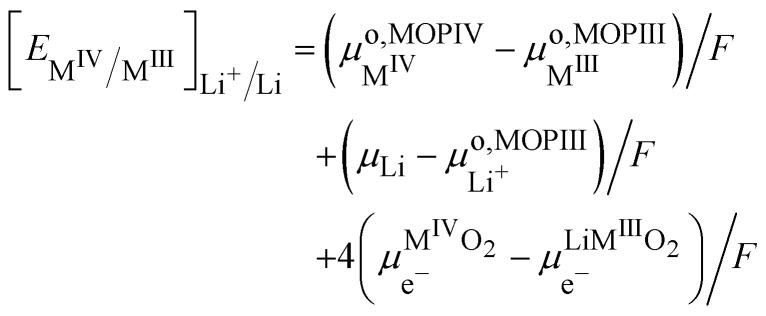
37
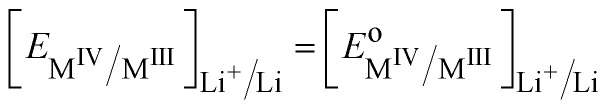
Eqn (37) shows that the Nernst potential is constant for ideally segregated phase and does not depend on the state-of-charge *x*.

It is important to realise that the standard redox potential for segregated systems 
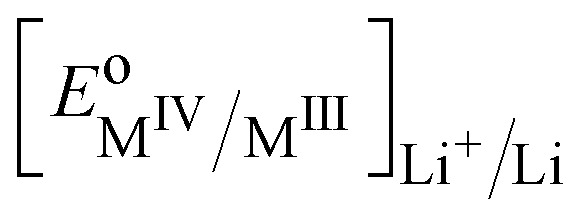
, differs from that for homogeneous systems given by eqn (23) and (24) as the standard states are different.

In the case of Li_*x*_CoO_2_, biphasic domains have been observed for 0.75 < *x* < 0.94 and a potential plateau shape of the discharge curves for this lithiation domain have been measured,^26^ as also shown in Fig. 11.

This equation is also corroborated in the case of lithium iron phosphate battery where the oxidized and reduced phases are segregated, as discussed earlier by Delmas *et al.* with the so-called domino cascade effect,^27^ and where the discharge curves are flat over a large domain, as shown in Fig. 11.

In the case of graphite also shown in Fig. 11, the presence of two plateaux suggests the formation of different segregated lithium/graphite phases.

## 5. Fermi level and electronic conductivity in a redox solid

### 5.1. Conductivity and electrochemical potential

Conductivity in a redox solid is related to the flux of charged species, namely the electrons and the mobile ions and stems from a gradient of electrochemical potential of the electron and the ion respectively. The flux of a species, *i*, is defined by

38 ***J***_*i*_ = −*c*_*i*_*ũ*_*i*_∇*μ̃*_*i*_

where *ũ*_*i*_ is the electrochemical mobility, which is always positive and where the electrochemical potential is given by

39 *μ̃*_*i*_ = *μ*^o^_*i*_ + *RT* ln *c*_*i*_ + *V̄*_*i*_*p* + *S̄T* + *z*_*i*_*Fϕ*

where *μ*^o^_*i*_ is the standard chemical potential, *V̄* and *S̄* are the partial molar volume and partial molar entropy, respectively.

The electric current density carried by the species *i*, ***j***_*i*_, is related to the flux and the electrical conductivity relates to the current density to a gradient of inner potential and therefore to the electric field **E**

40 ***j***_*i*_ = *z*_*i*_*F****J***_*i*_ = −*σ*_*i*_∇*ϕ* = *σ*_*i*_**E**

where *σ*_*i*_ is the electrical conductivity. In the case of a metal oxide, we should consider both the electronic *σ*_e_ and the ionic *σ*_Li^+^_. The overall conductivity is the sum of the electronic and ionic conductivity, which by considering the electroneutrality of the MOP particle should be equal.

### 5.2. Dry MOP materials

**Fig. 13:**
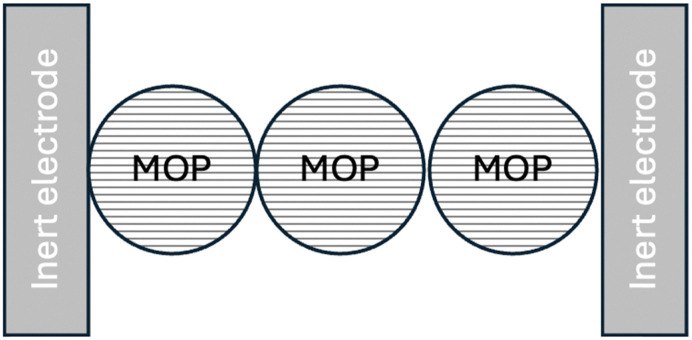
Dry case conductivity. Electron transfer reactions occur at the inert electrode/MOP interface.

As shown in Fig. 13, we shall first distinguish dry MOP particles located between two inert electrodes, sometimes called ion blocking electrodes.

Here, using high frequency AC perturbations, a potential difference is applied between the two inert electrodes where electron transfer reactions take place, and the resistance is measured as a function of temperature to calculate the activation energy. Considering that the electric current through a partially or fully lithiated particle has two components: electron hopping between redox sites, with an activation energy, *E*_a_, related to the lattice re-organisation energy, *λ*_L_, (*E*_a_ = *λ*_L_/4 as shown in the ESI† in Fig. A4) which includes the motion of the lithium ions.

The lattice re-organisation energy can be calculated by the Wiedemann–Franz law for electron hopping between redox sites.^28^41
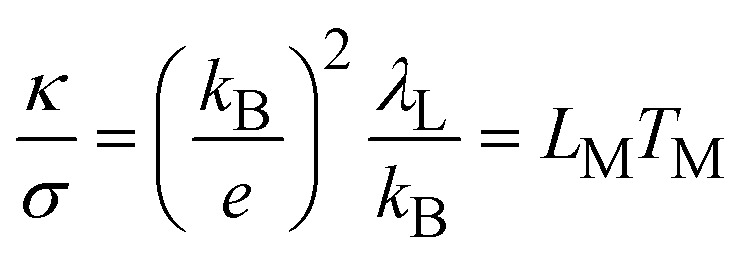
where *κ* is the thermal conductivity, *σ* the electrical conductivity and *k*_B_ the Boltzmann constant. *L*_M_ is the Lorenz number for electron hopping and *T*_M_ the effective temperature associated to *λ*_L_. In the classical Wiedemann–Franz law for electrical conductivity in metals, the ratio of thermal and electrical conductivities reads42
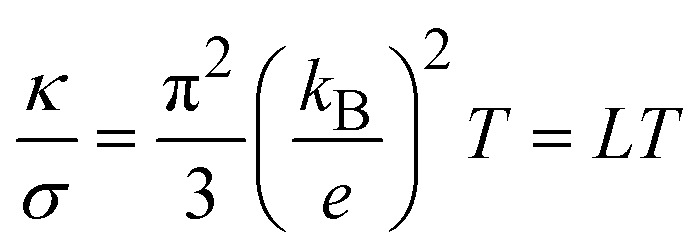
as both conductivities are associated to the motion of quasi-free charge-carrying particles, namely the electrons. In eqn (41), *L* is the classical Lorenz number.

Electron hopping occurs not only inside the metal oxide particle but also between the particles themselves. Literature tends to suggest that the activation energies for inter-particles conduction are largely dependent on the sample preparation conditions.

In the limiting case of intragrain electron hopping between fixed sites, the system is similar to a redox polymer, and it behaves as if the redox centres were diffusing with an apparent diffusion coefficient *D*_e_.

43 *D*_e_ = *k*_ex_*c*^tot^_redox_Δ*x*^2^

where *k*_ex_ is the isotopic rate constant for electron hopping, the *c*^tot^_redox_ the concentration of redox centres and Δ*x* the mean distance of hopping between the redox centres.^29^

Savéant developed further the theory for coupling equivalent diffusion and migration laws^30^ and shows that the equivalent flux of reduced centres could be written as:44
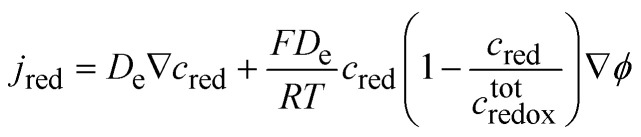
We can define, using the Einstein relation, the electrochemical mobility of the electron *ũ*_e_ as

45 *D*_e_ = *RTũ*_e_

**Fig. 14:**
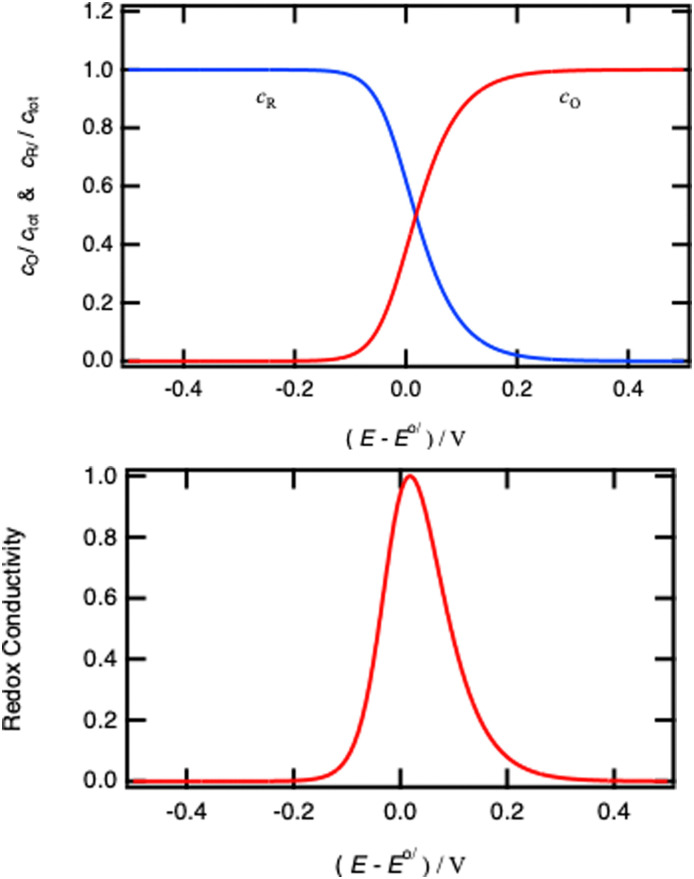
Normalised concentrations using eqn (26) and normalised intraparticle redox conductivity from eqn (45).

If the phase is ideally homogeneous there is no concentration gradient, and the redox conductivity is given by46
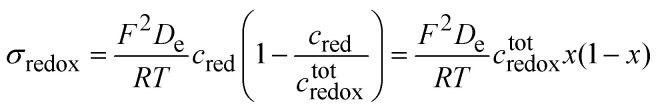
using the definition of *x* given by eqn (25). The potential dependence of the redox conductivity is given by solving the Nernst eqn (26) for *x* as shown in Fig. 14.

When comparing Fig. 4 and 14, one can notice that the different potential dependence of the concentrations as the standard potential corresponds to *x* = 0.618. Of course, this model assumes fast lithium transport and the overall intragrain conductivity may be attenuated by a slow ionic motion. Indeed, redox polymers are usually in solutions and electroneutrality is ensured by the electrolyte.

For Li_*x*_CoO_2_, we can distinguish the electronic conductivity of the fully lithiated material from that of the partially oxidised one. Indeed, the electric conductivity of Li_*x*_CoO_2_ was shown, as early as 1989, to depend on the degree of lithiation,^31^ the fully lithiated material behaving like a semi-conductor with an activated electron transport mechanism (activation energy = 0.33 eV for *x* = 1), and the material becoming more “metallic” with lithium extrusion. The change of conductivity was suggested to be due variations in the Fermi level, which in fact is the onset of the redox conductivity.

Actually, in the same way that the electrical conductivity depends on the degree of lithiation, the thermal conductivity of Li_*x*_CoO_2_, can be reversibly electrochemically modulated over a considerable range from Li_1.0_CoO_2_ to Li_0.6_CoO_2_, in a system which has only a 1.3% change in the unit cell volume.^32^

All in all, the model above shows the importance of redox conductivity when homogeneous domains are present in a material, and clearly show the variation of redox conductivity with the Nernst potential. Near the standard potential, the material has the highest conductivity and the lowest when fully oxidised or reduced.

In segregated materials, the redox species are either fully oxidised or reduced and redox conductivity does not occur. This is a reason why lithium iron phosphate is a poor electronic conductor that needs to be carbon coated to ensure electronic conductivity.

### 5.3. Wet MOP material in a lithium electrolyte

**Fig. 15:**
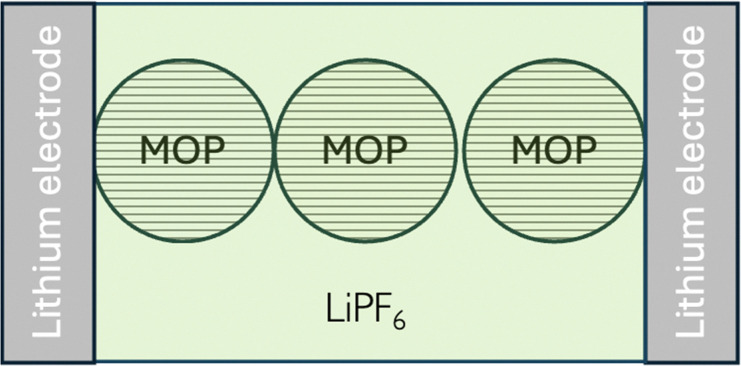
Wet case conductivity. Electron transfer reactions occur at the lithium electrode/MOP interface, where lithium transfer reactions occur both at the lithium electrode/MOP and lithium electrode/electrolyte interfaces.

When the metal oxide particles are immersed in an electrolyte solution containing lithium cations as shown in Fig. 15 located in a symmetrical cell with reversible lithium metal electrodes, the redox conductivity discussed above combined with the inner lithium conductivity will still occur. In parallel, we also have the ionic conductivity of the electrolyte solution.

Another conductivity that could be considered is “surface hopping”. As discussed above, one way to lower the Fermi level is to charge the metal oxide particle by oxidation at its surface, as shown in Fig. 8. Here, electron hopping between surface sites will depend on two re-organisation energies. A solvent reorganisation energy associated to the adjacent electrolyte, which will depend on the polarity of the solvent and the ionic strength and a lattice reorganisation energy which does not include lithium-ion insertion or extrusion and is therefore likely to be small. Again, this surface conductivity will be of a redox nature and maximum when both M^IV^ and M^III^ are present at the surface, and low when the interface is fully reduced or oxidised.

## 6. Applications to commercial electrochemical storage system

All concepts discussed above are keys to understand electrochemical storage systems and define their electrochemical properties. Unfortunately, the application of such laws is not straightforward since most of them can be applied to perfect systems referred generally to thin film materials having no defect, or almost not defect.^33^ Furthermore, in thin film model material, there is no conductive carbon needed nor any binder thus limiting the number of interfaces, strictly limited to the interaction between the electrolyte (liquid, solid or polymer) and the electroactive materials.^34^ Real electrochemical systems (except for micro-batteries) are far away from any ideal system since the electrode materials rely on bulky electroactive materials in contact with a binder and a conductive agent. The electrode materials are thus porous allowing the embedding with electrolyte to ensure ionic conductivity, whereas current collector, conductive agent and sometimes electroactive materials (if they are electronic conductor or semi-conductors) guarantee the electronic pathway.^35–37^ However, at this stage, it is difficult to control both electronic/ionic pathways perfectly in 3D in an electrode generating then several interfaces, each of them having an electrochemical signature that could then influence the overall electrochemical response which is finally a sum of all possible processes occurring at each of the interfaces.^38^

Considering these facts, several points should be considered, among them the native surface layer developed at the surface of the electroactive materials (like Li_2_CO_3_, LiOH, *etc.*),^39,40^ that can then take part to the electrochemical activities, influencing the Fermi level as well as the solvation shell around the particles. It could also influence the contact potential and process like a biphasic reaction, the shell being one phase and the core another one in case the surface layer is electrochemically active.

Similarly for the contact potential and the Fermi level, carbon coating or coatings in general are used to enhance the electronic conductivity of the electroactive materials or to protected them from enhance decomposition (chemical one).^41^ Thus, and due to the very thin dimension of the coating, the Fermi level of the electron coming from the coating varies to follow that of the contact electrode by changing its electronic surface charge and hence its outer potential. A typical example here, is the reaction of LiFePO_4_ material which is carbon coated to ensure proper electrochemical reaction and where carbon coating is following the potential of the Fe^2+^/Fe^3+^ potential.^42^

Also, the contact potential between two phases (segregated system or biphasic domain), can be influenced by the particle size and particle geometry.^43,44^ As an example, the reaction mechanism between a single particle and a polycrystalline material should be strictly identical. However, depending on the charge transfer process and depending on the solid-state diffusion, biphasic behaviour can occur in large particles, especially is defect are present, whereas small particles would react through solid solution process.^45,46^ Silicon material and LFP are good example is both cases, as their reaction pathways are similar, alloying reaction through core–shell process for the former and biphasic reaction for the latter. Both suffer very low electronic conductivity, that is why, nanoparticles are generally preferred to decrease the solid-state diffusion length, and they are both carbon-coated to ensure a proper electrochemical activity. Furthermore, the reaction mechanism of LFP is influenced by the cycling rate behaving through solid solution close to thermodynamic condition (slow cycling rate) and through solid solution when the cycling rate is high.^47,48^ As for silicon, such difference is difficult to investigate since Si is undertaking an alloying reaction.

## 7. Novel electrochemical storage system

As the field of batteries is constantly evolving, novel type of materials emerged which are currently under investigation at R&D level.

As an example, conversion-based materials and alloying type materials are widely investigated in the battery field as negative electrode materials but to date their commercialization remain impaired by their large volume changes generating fractures and their uncontrolled SEI.^49,50^ Their redox mechanism is still described in this paper since they react through a core–shell process.^51–56^ At the positive electrode material, we can also investigate more exotic materials such as the conversion type materials mainly FeF_2_ & FeF_3_, the vanadium phosphate and the lithium rich material where not only the transition metal is redox active but also the oxygen, leading to the so-called anionic redox center, again in those cases, the materials behave either as a core–shell process or through a solid solution,^57,58^ both described in the paper.

Generally, at the industrial level, the materials employed are more conventional and rely mostly on solid solution reaction, since these reactions are better controlled from a morphological point of view, their volume changes being rather friendly during redox mechanisms. Recently, a shift was seeing at the industrial scale with the development of blend of graphite negative electrode mixed with a small percentage of silicon nanoparticles (5 to 10 wt%) to enhance the energy density of the negative electrode. Currently, the fading observed in those system is more important than the one of graphite used solely, the reason being the difference in volume changes between both materials, and the SEI that covers the Si nanoparticles.^59,60^

For Li–S batteries, where there are two different issues, the first one is the complexity of the Li metal negative electrode (described in eqn (18)), and the second one is the dissolution of sulfur material into the electrolyte and the re-crystallization afterwards into insulating Li_2_S material that pollutes the Li metal negative electrode and generate the so call polysulfides shuttle. Again, this mechanism is so particular that it is difficult yet to address it through this paper.

For the organic electrode materials, where the mechanism is also extremely complex, and yet still debated,^61^ the absorption behavior couple to possible anionic redox reaction and novel chemical bounding cannot be excluded but can be thermodynamically treated similarly to derivation of eqn (26).

In a similar manner, other chemistry types such as Na, Mg, K, Al type batteries are intensively investigated, and except Na-ion batteries, there are doubts that the others will be commercialized one day. But to date, even in novel chemistry type, the reaction mechanisms remain the same, either a solid solution or a biphasic reaction. Finally, a particular attention should be made here, that all reaction mechanism described are involving a liquid electrolyte solution. However, more and more researchers tend to replace a liquid electrolyte by a solid one, thus having two solids close by to react, stressing the point of interface mechanisms and redox mechanisms.

## Conclusions

All in all, we have reviewed here the redox properties of positive electrode materials in lithium-ion batteries and considered two ideal systems. The homogenous redox solid where the oxidised and reduced metal centres are randomly distributed in the metal oxide particles and the segregated redox solid where they are physically separated. Two different Nernst equations were obtained. In the former case, where both M^IV^ and M^III^ are present, the concept of redox conductivity, as introduced to treat the conductivity of redox polymers, can be applied. In the case of biphasic materials in contact, this redox conductivity does not apply, and the materials are poorer electronic conductors.

Similar thermodynamic considerations can be applied for other systems such as organic cathodes, sulfide cathodes and conversion materials or anion intercalation materials. This requires understanding of the phase changes in the materials during redox reactions. If no segregation takes place, Nernst equations can be derived similarly to eqn (26). If segregation and multiple phases are present, a treatment similar to Section 4.6 is required. Materials with multiple phases (for example graphite) and multiple redox sites at different potentials require their own treatment, and will be a topic of a follow-up paper.

We believe that further work on understanding of the thermodynamics of different phases as well as activities of the solid species is required to be able to better explain the experimental curves.

## Author contributions

Conceptualization, formal analysis, writing – original draft (PP, CV, HHG), funding acquisition (PP), visualization (PP, HHG).

## Data availability

No primary research results, software or code have been included and no new data were generated or analysed as part of this perspective.

## Conflicts of interest

There are no conflicts to declare.

## Supplementary Material

EE-018-D4EE04560B-s001
