# Supplementary material for: The redox aspects of lithium-ion batteries
Source: Energy Environ Sci. 2024 Dec 14;18(4):1658–72. doi: 10.1039/d4ee04560b (PMC11753199; doi:10.1039/d4ee04560b)
Supplement: EE-018-D4EE04560B-s001 [file EE-018-D4EE04560B-s001.pdf]

Supplementary Information (SI) for Energy & Environmental Science.
This journal is © The Royal Society of Chemistry 2024

Electrical Supplementary Information

The redox aspects of lithium-ion batteries

Pekka Peljo[1], Claire Villevieille[2] and Hubert H. Girault[3,4]

1- Research Group of Battery Materials and Technologies, Department of Mechanical and Materials Engineering, University of Turku, FI-20014 Turun Yliopisto, Finland
2. LEPMI, Univ. Grenoble Alpes, Univ. Savoie Mont Blanc, CNRS, Grenoble INP, LEPMI, Grenoble, France
3. Institute of Chemical Science and Engineering, Station 6, Ecole Polytechnique Federale de Lausanne, CH-1015 Lausanne, Switzerland
4. Material Science and Nanoengineering (MSN) department, University Mohammed VI Polytechnic, 43 150 Ben Guerir, Morocco

This ESI is based on the textbook of H. Girault.[1]

A1. Electrochemical potential of electron in solution in the presence of a redox couple

In view of its extreme reactivity, the electron can exist in solution as a solvated species only for relatively short periods of time, and consequently we will not deal with the case of the electrochemical potential of the solvated electron. Nevertheless, it can sometimes be useful to use the rather abstract notion of electrochemical potential or even the notion of the Fermi level of the electron in solution, knowing that the electron resides on a reduced species. To better understand this concept of a Fermi level for a redox pair in solution, we will consider the example of the redox pair Fe^{3+}/Fe^{2+}.

To define the actual chemical potential of the electron in solution, consider the oxidation of $Fe^{2+}_{(aq)}$ to $Fe^{3+}_{(aq)}$ in solution and consider the electron as a species in its own right

$$Fe^{2+}_{(aq)} \rightleftarrows Fe^{3+}_{(aq)} + e^{-}_{(aq)}$$

From a thermodynamic point of view, we can treat this virtual equilibrium and write

$$\tilde{m}^{S}_{Fe^{2+}} = \tilde{m}^{S}_{Fe^{3+}} + \tilde{m}^{S}_{e^{-}} \qquad \text{(A1)}$$

thus defining the electrochemical potential of the electron in solution.

As shown in fig. A1, we can deconvolute this oxidation by using the following steps:

- $Fe^{2+}_{(aq)}$ removal from the aqueous phase. The corresponding work is the opposite of the electrochemical potential of the Fe^{2+} ion,

[1] Translated and adapted from H.H. Girault Electrochimie Physique et Analytique, 2nd Edition, 2007, EPFL Press

$$\tilde{\mu}^{S}_{Fe^{2+}} = \alpha^{S}_{Fe^{2+}} + 2F\psi^{S} \quad \text{(A2)}$$

• Fe^{3+} addition to the aqueous phase. The work corresponds to the electrochemical potential of the Fe^{3+} ion

$$\tilde{\mu}^{S}_{Fe^{3+}} = \alpha^{S}_{Fe^{3+}} + 3F\psi^{S} \quad \text{(A3)}$$

• Transfer of the electron from vacuum to the solution. The work corresponds to the electrochemical potential of the electron in solution

$$\tilde{\mu}^{S}_{e^{-}} = \alpha^{S}_{e^{-}} - F\psi^{S} \quad \text{(A4)}$$

Thus, at equilibrium, we can define the real chemical potential of the electron in solution by

$$\alpha^{S}_{e^{-}} = \alpha^{S}_{Fe^{2+}} - \alpha^{S}_{Fe^{3+}} \quad \text{(A5)}$$

More generally, for a one electron redox pair , we can write

$$\alpha^{S}_{e^{-}} = \alpha^{S}_{red} - \alpha^{S}_{ox} \quad \text{(A6)}$$

and define the standard real chemical potential of the electron in solution by

$$\alpha^{\ominus,S}_{e^{-}} = \alpha^{\ominus,S}_{red} - \alpha^{\ominus,S}_{ox} \quad \text{(A7)}$$

The notion of the real chemical potential of the electron in solution therefore depends on the nature of the redox couple. The actual chemical potential corresponds to the work accompanying the transfer of an electron from infinity to an uncharged solution, as shown in figure A1. By analogy with metals, the actual chemical potential of the electron in solution is the opposite of the work of extracting an electron from a solution. Thus, we can define an oxidation energy ΔE_O for an oxidation reaction in solution but considering the electron as a species at rest in vacuum.

$$Fe^{2+}_{(aq)} \longrightarrow Fe^{3+}_{(aq)} + e^{-}_{(v)}$$

For one mole, we can the write

$$\Delta E_O = -\tilde{\mu}^{S}_{e^{-}} \quad \text{(A8)}$$

or again in the example in figure A1 assuming that the solution is ideally diluted

$$\Delta E_O = \tilde{\mu}^{S}_{Fe^{3+}} - \tilde{\mu}^{S}_{Fe^{2+}} = \alpha^{\ominus}_{Fe^{3+}} - \alpha^{\ominus}_{Fe^{2+}} + RT\ln\left(\frac{c_{Fe^{3+}}}{c_{Fe^{2+}}}\right) + F\psi^{S} \quad \text{(A9)}$$

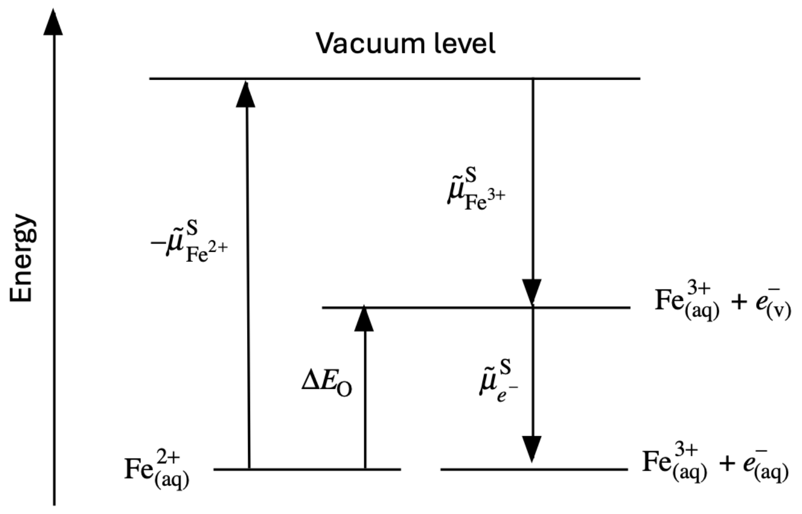

Fig. A1: Electrochemical potential of the electron in solution in the presence of a redox pair

A2. Fermi level for the redox pair Fe^{3+}/Fe^{2+} in solution

In solution, the Fe^{3+} and Fe^{2+} ions are hydrated and the interaction of these ions with the solvent molecules must be considered. Thus, the presence of the solvent has the effect of lowering the energy levels of the oxidized and reduced species, compared to their respective levels in the gas phase which define the ionization energy. For an uncharged liquid phase, the difference in energy related to the solvation of an ion is equal to the hydration Gibbs energy ΔG_{hyd}.

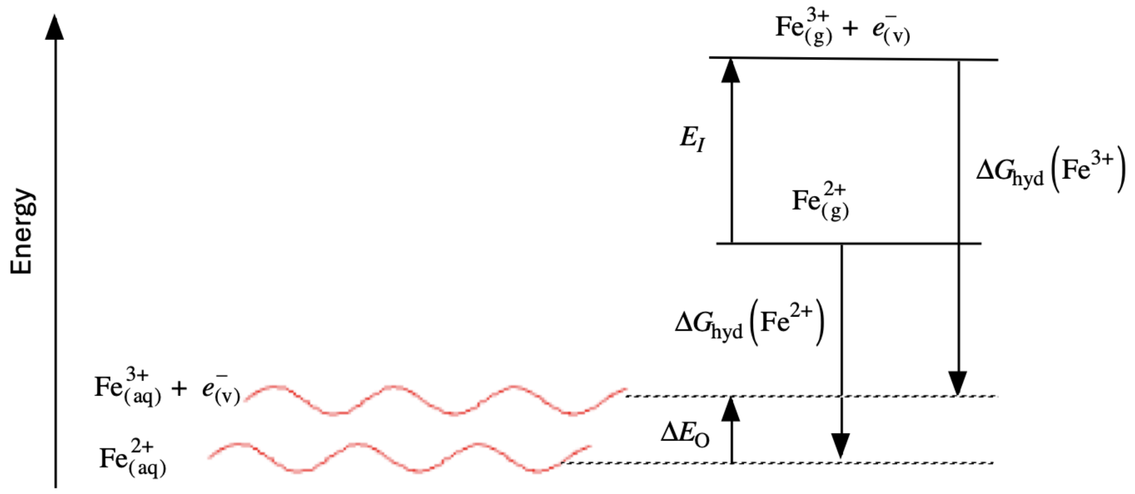

Fig. A2 Energies of ions in solution and in the gas phase. E_I stands for the ionization energy in the gas phase.

The main difference between an aqueous solution and the gas phase is due to the fact that the polarization energy of the polar solvent fluctuates with molecular agitation, as shown

in figure A2. Thus, the oxidation energy in solution defined by the equation A8 also fluctuates with the polarization of the solvent, unlike the ionization energy E_I which is an intrinsic property of the redox couple in the gas phase.

As a first approximation, we can assume that the solvation energy varies harmonically with the polarization of the solvent, as illustrated by a parabola in figure A3. To be more rigorous, coordinates associated with normal modes should be considered for all degrees of freedom involved in solvent fluctuation. In this case, the energy curves would be paraboloids. Nevertheless, we will be satisfied here with a system with a single coordinate that we call the polarization of the solvent.

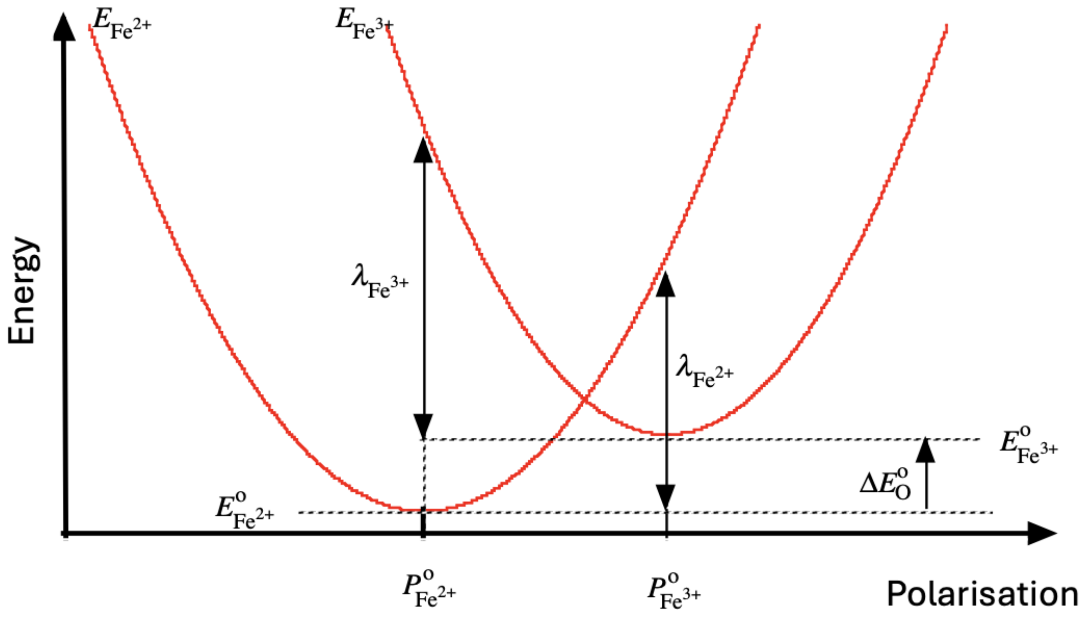

Fig. A3 Variation in energy levels with fluctuation in solvent polarization.

We can thus write that the variation in energy related to the fluctuation of the solvent for the solvated Fe^{2+} ion is written as

$$E_{Fe^{2+}} - E^o_{Fe^{2+}} = \frac{K_{Fe^{2+}}}{2}\left(P - P^o_{Fe^{2+}}\right)^2 \quad \text{(A10)}$$

and that for the solvated Fe^{3+} ion

$$E_{Fe^{3+}} - E^o_{Fe^{3+}} = \frac{K_{Fe^{3+}}}{2}\left(P - P^o_{Fe^{3+}}\right)^2 \quad \text{(A11)}$$

or

$$E_{Fe^{3+}} - E^o_{Fe^{2+}} = \frac{K_{Fe^{3+}}}{2}\left(P - P^o_{Fe^{3+}}\right)^2 + \Delta E^o_O \quad \text{(A12)}$$

where $K_{Fe^{x+}}$ is the curvature of the parabola, *P is* the polarisation of the solvent out of equilibrium, $P^0_{Fe^{x+}}$ the optimal polarization of the solvent around the Fe^{2+} ion, and where ΔE^o_O is the oxidation energy defined by the equation (A8) when the polarizations are optimal.

$$\Delta E_{O}^{0} = E_{Fe^{3+}}^{0} - E_{Fe^{2+}}^{0} \quad \text{(A13)}$$

To determine the curvature of the parabolas, let us compare their relative position. By introducing the parameter $\lambda_{Fe^{2+}}$ defined by

$$\lambda_{Fe^{2+}} = \frac{K_{Fe^{2+}}}{2}\left(P_{Fe^{3+}}^{o} - P_{Fe^{2+}}^{o}\right)^{2} \quad \text{(A14)}$$

the equation (A10) for Fe^{2+} is written

$$E_{Fe^{2+}} - E_{Fe^{2+}}^{o} = \frac{\lambda_{Fe^{2+}}\left(P - P_{Fe^{2+}}^{o}\right)^{2}}{\left(P_{Fe^{3+}}^{o} - P_{Fe^{2+}}^{o}\right)^{2}} \quad \text{(A15)}$$

So when $P = P_{Fe^{3+}}^{0}$, then

$$E_{Fe^{2+}} - E_{Fe^{2+}}^{o}\Big|_{P=P_{Fe^{3+}}^{o}} = \lambda_{Fe^{2+}} \quad \text{(A16)}$$

This equation allows us to give a physical meaning to the parameter $\lambda_{Fe^{2+}}$. Indeed, $\lambda_{Fe^{2+}}$ corresponds to the energy of re-organization of the solvent following the very rapid reduction of Fe^{3+} to Fe^{2+} (fast compared to the solvation time scale), where we end up with an Fe^{2+} ion having the optimal solvation of an Fe^{3+} ion. $\lambda_{Fe^{2+}}$ corresponds to the relaxation energy of the solvent from this out-of-equilibrium state to the optimal polarization.

In the same way, for Fe^{3+}, we can set the parameter $\lambda_{Fe^{3+}}$

$$\lambda_{Fe^{3+}} = \frac{K_{Fe^{3+}}}{2}\left(P_{Fe^{3+}}^{o} - P_{Fe^{2+}}^{o}\right)^{2} \quad \text{(A17)}$$

and the equation (A12) becomes

$$E_{Fe^{3+}} - E_{Fe^{3+}}^{o} = \frac{\lambda_{Fe^{3+}}\left(P - P_{Fe^{3+}}^{o}\right)^{2}}{\left(P_{Fe^{3+}}^{o} - P_{Fe^{2+}}^{o}\right)^{2}} \quad \text{(A18)}$$

when $P = P_{Fe^{2+}}^{0}$, then

$$E_{Fe^{3+}} - E_{Fe^{3+}}^{o}\Big|_{P=P_{Fe^{2+}}^{o}} = \lambda_{Fe^{3+}} \quad \text{(A19)}$$

Again, $\lambda_{Fe^{3+}}$ corresponds to the energy of re-organization of the solvent following the very rapid oxidation of Fe^{2+} into Fe^{3+}, where we end up with an Fe^{3+} ion having the optimal solvation of an Fe^{2+} ion.

Rather than plotting the fluctuation of an energy level as a function of polarization as shown in figure A3, it can also be plotted as a function of the difference in the ΔE_{O} energies of the oxidized and reduced states, which represents the oxidation energy in solution for a given polarization (with electron extraction to infinity), i.e., the vertical passage from one parabola to another.

$$\Delta E_{O} = E_{Fe^{3+}} - E_{Fe^{2+}} \quad \text{(A20)}$$

By difference of the equations (A18) and(A15), we obtain

$$\Delta E_{\mathrm{O}} - \Delta E_{\mathrm{O}}^{\mathrm{o}} = \frac{\lambda_{\mathrm{Fe}^{3+}}\left(P - P_{\mathrm{Fe}^{3+}}^{\mathrm{o}}\right)^2 - \lambda_{\mathrm{Fe}^{2+}}\left(P - P_{\mathrm{Fe}^{2+}}^{\mathrm{o}}\right)^2}{\left(P_{\mathrm{Fe}^{3+}}^{\mathrm{o}} - P_{\mathrm{Fe}^{2+}}^{\mathrm{o}}\right)^2} \qquad \text{(A21)}$$

For simplicity, let us make the additional assumption that the re-organization energies are equal, *i.e.* that the two parabolas in figure A3 have the same curvature. In this case, the equation (A21) becomes

$$\Delta E_{\mathrm{O}} - \Delta E_{\mathrm{O}}^{\mathrm{o}} = \lambda\left(\frac{P_{\mathrm{Fe}^{2+}}^{\mathrm{o}} + P_{\mathrm{Fe}^{3+}}^{\mathrm{o}} - 2P}{P_{\mathrm{Fe}^{3+}}^{\mathrm{o}} - P_{\mathrm{Fe}^{2+}}^{\mathrm{o}}}\right) \qquad \text{(A22)}$$

By substitution, the energy fluctuation for Fe^{2+} given by the equation (A15) becomes

$$E_{\mathrm{Fe}^{2+}} - E_{\mathrm{Fe}^{2+}}^{\mathrm{o}} = \frac{\left(\Delta E_{\mathrm{O}} - \Delta E_{\mathrm{O}}^{\mathrm{o}} - \lambda\right)^2}{4\lambda} \qquad \text{(A23)}$$

and the one for Fe^{3+} given by the equation (A18) is written

$$E_{\mathrm{Fe}^{3+}} - E_{\mathrm{Fe}^{3+}}^{\mathrm{o}} = \frac{\left(\Delta E_{\mathrm{O}} - \Delta E_{\mathrm{O}}^{\mathrm{o}} + \lambda\right)^2}{4\lambda} \qquad \text{(A24)}$$

Figure A4 illustrates that fluctuations in the energy of ions in solution also vary parabolically with the fluctuation of oxidation energy. $\Delta E_{\mathrm{O}} - \Delta E_{\mathrm{O}}^{\mathrm{o}}$ When $\Delta E_{\mathrm{O}} = \Delta E_{\mathrm{O}}^{\mathrm{o}} + \lambda$, The reduced species is in its optimal polarization state and when $\Delta E_{\mathrm{O}} = \Delta E_{\mathrm{O}}^{\mathrm{o}} - \lambda$ the oxidized species is in its optimal polarization state.

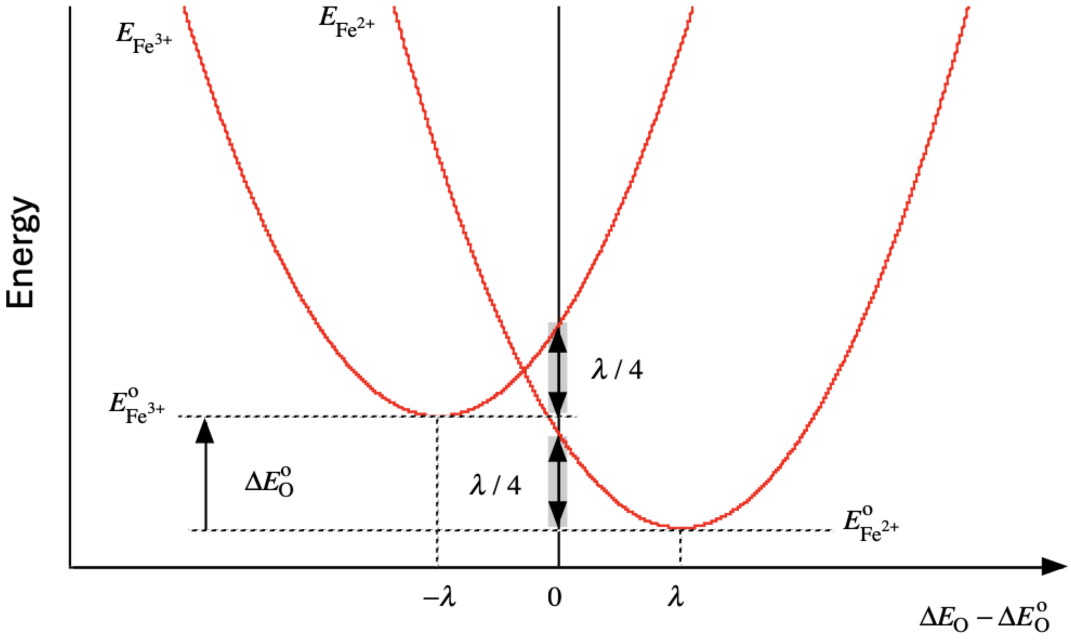

Fig. A4 Variations in the energy levels of oxidized and reduced species with solvent fluctuation given by equations (A23) and (A24).

We can hypothesize that the fluctuations in energy levels $E_{Fe^{2+}}$ and $E_{Fe^{3+}}$ follow a Boltzmann statistic that can be written as a function of the energy difference associated with the fluctuation of the solvent around the ion.

The normalized distribution function of the energy level $E_{Fe^{2+}}$, which represents the probability density of the energy level of the reduced species, is then written

$$W_{Fe^{2+}}(\Delta E_O) = \frac{f(E_{Fe^{2+}})}{\int_0^\infty f(E_{Fe^{2+}})\mathrm{d}E_{Fe^{2+}}} = \frac{e^{-\left(E_{Fe^{2+}} - E^o_{Fe^{2+}}\right)/kT}}{\int_0^\infty e^{-\left(E_{Fe^{2+}} - E^o_{Fe^{2+}}\right)/kT}\mathrm{d}E_{Fe^{2+}}}$$

$$= \frac{e^{-\frac{\left(\Delta E_O - \Delta E^o_O - \lambda\right)^2}{4kT\lambda}}}{\int_{-\infty}^{\infty} e^{-\frac{\left(\Delta E_O - \Delta E^o_O - \lambda\right)^2}{4kT\lambda}}\mathrm{d}\Delta E_O} = \frac{e^{-\frac{\left(\Delta E_O - \left(\Delta E^o_O + \lambda\right)\right)^2}{4kT\lambda}}}{\sqrt{4\pi kT\lambda}} \qquad \text{(A25)}$$

and the one for Fe^{3+}

$$W_{Fe^{3+}}(\Delta E_O) = \frac{f(E_{Fe^{3+}})}{\int_0^\infty f(E_{Fe^{3+}})\mathrm{d}E_{Fe^{3+}}} = \frac{e^{-\left(E_{Fe^{3+}} - E^o_{Fe^{3+}}\right)/kT}}{\int_0^\infty e^{-\left(E_{Fe^{3+}} - E^o_{Fe^{3+}}\right)/kT}\mathrm{d}E_{Fe^{3+}}}$$

$$= \frac{e^{-\frac{\left(\Delta E_O - \Delta E^o_O + \lambda\right)^2}{4kT\lambda}}}{\int_{-\infty}^{\infty} e^{-\frac{\left(\Delta E_O - \Delta E^o_O + \lambda\right)^2}{4kT\lambda}}\mathrm{d}\Delta E_O} = \frac{e^{-\frac{\left(\Delta E_O - \left(\Delta E^o_O - \lambda\right)\right)^2}{4kT\lambda}}}{\sqrt{4\pi kT\lambda}} \qquad \text{(A26)}$$

These normalized distribution functions can be plotted as representing the energy probability densities for oxidized and reduced species as a function of the oxidation energy in solution ΔE_O, as shown in figure A5. The curves thus obtained are Gaussians. It is important to understand that each Gaussian distribution does not represent a band of several energy levels, but rather the fluctuation of a single level associated with the fluctuation of the polarization of the solvent.

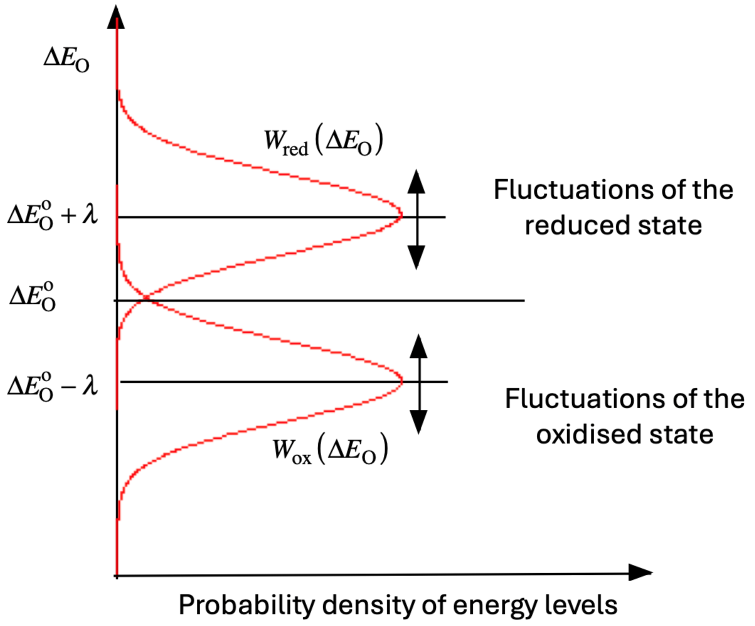

Fig. A5 Distribution functions of the energy states of the reduced species (level occupied by the electron) and the oxidized species (vacant level)

When the oxidation energy ΔE_O is equal to ΔE_O^o, *i.e.,* the oxidation energy when the oxidized state and the reduced state are in their optimal solvation state (see figure A5), the distributions functions $W_{Fe^{2+}}(\Delta E_O)$ and $W_{Fe^{3+}}(\Delta E_O)$ are equal. As illustrated in figure A4, the deviations of the solvation energies from the minima are then also equal.

$$E_{Fe^{3+}} - E^0_{Fe^{3+}} = E_{Fe^{2+}} - E^0_{Fe^{2+}} = \lambda / 4 \qquad \text{(A27)}$$

The densities of energy states of redox species are obtained by considering their concentrations and the probability of the existence of this state

$$D_{Fe^{2+}}(\Delta E_O) = c_{Fe^{2+}} W_{Fe^{2+}}(\Delta E_O) \qquad \text{(A28)}$$

and

$$D_{Fe^{3+}}(\Delta E_O) = c_{Fe^{3+}} W_{Fe^{3+}}(\Delta E_O) \qquad \text{(A29)}$$

The densities of energy states of redox species are obtained by considering their concentrations and the probability of the existence of this state

$$D_{Fe^{2+}}(\Delta E_O) = D(\Delta E_O) \cdot (1 - F(\Delta E_O)) \qquad \text{(A30)}$$

Taking the ratio of the equations (A29) and (A30), we obtain

$$\frac{1-F(\Delta E_O)}{F(\Delta E_O)} = \frac{D_{Fe^{2+}}(\Delta E_O)}{D_{Fe^{3+}}(\Delta E_O)} = \frac{c_{Fe^{2+}}W_{Fe^{2+}}(\Delta E_O)}{c_{Fe^{3+}}W_{Fe^{3+}}(\Delta E_O)}$$

$$= \frac{c_{Fe^{2+}}e^{-\frac{\left(\Delta E_O-\left(\Delta E_O^o+\lambda\right)\right)^2}{4kT\lambda}}}{c_{Fe^{3+}}e^{-\frac{\left(\Delta E_O-\left(\Delta E_O^o-\lambda\right)\right)^2}{4kT\lambda}}} = \frac{c_{Fe^{2+}}e^{\frac{\Delta E_O-\Delta E_O^o}{kT}}}{c_{Fe^{3+}}} = e^{\frac{\Delta E_O-\Delta E_{F_O}}{kT}} \quad \text{(A31)}$$

by defining

$$\Delta E_{F_O} = \Delta E_O^o + kT\ln\left(\frac{c_{Fe^{3+}}}{c_{Fe^{2+}}}\right) \quad \text{(A32)}$$

Thus, the probability $F(\Delta E_O)$ that the redox couple is oxidized obtained from the equation (A31) is in fact a Fermi-Dirac distribution with two energy levels

$$F(\Delta E_O) = \frac{1}{1+e^{\frac{\Delta E_O-\Delta E_{F_O}}{kT}}} \quad \text{(A33)}$$

where ΔE_{F_O} is the Fermi energy for this distribution. This energy represents the oxidation energy for which the densities of energy states $D_{Fe^{2+}}\left(\Delta E_O\right)$ and $D_{Fe^{3+}}\left(\Delta E_O\right)$ are equal. We thus deduce that for this energy we have

$$\frac{c_{Fe^{3+}}}{c_{Fe^{2+}}} = e^{\frac{\Delta E_{F_O}-\Delta E_O^o}{kT}} \quad \text{(A34)}$$

In general, for example in the case of a metal, it is customary to visualize the filled energy states under the empty states. To do this, we can trace figure A5 and plot the density of the energy states no longer as a function of ΔE_O but as a function of the opposite ΔE_R defined by

$$\Delta E_R = -\Delta E_O = E_{Fe^{2+}} - E_{Fe^{3+}} \quad \text{(A35)}$$

The probability densities are then expressed

$$W_{Fe^{2+}}(\Delta E_R) = \frac{e^{-\frac{\left(\Delta E_R-\left(\Delta E_R^o-\lambda\right)\right)^2}{4kT\lambda}}}{\sqrt{4\pi kT\lambda}} \quad \text{(A36)}$$

and

$$W_{Fe^{3+}}(\Delta E_R) = \frac{e^{-\frac{\left(\Delta E_R-\left(\Delta E_R^o+\lambda\right)\right)^2}{4kT\lambda}}}{\sqrt{4\pi kT\lambda}} \quad \text{(A37)}$$

as shown in figure A6.

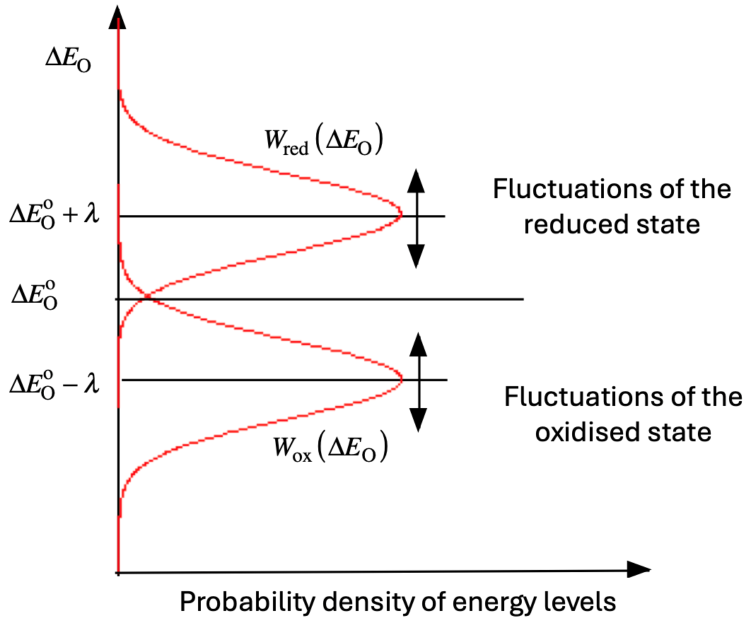

Fig. A6 Distribution functions of the energy states of the reduced species (level occupied by the electron) and the oxidized species (vacant level)

In the same way, the densities of energy states of redox species are obtained by considering their concentrations and the probability of the existence of this state

$$D_{\mathrm{Fe}^{2+}}(\Delta E_{\mathrm{R}}) = c_{\mathrm{Fe}^{2+}} W_{\mathrm{Fe}^{2+}}(\Delta E_{\mathrm{R}}) \qquad \text{(A38)}$$

and

$$D_{\mathrm{Fe}^{3+}}(\Delta E_{\mathrm{R}}) = c_{\mathrm{Fe}^{3+}} W_{\mathrm{Fe}^{3+}}(\Delta E_{\mathrm{R}}) \qquad \text{(A39)}$$

If $D(\Delta E_{\mathrm{R}})$ represents the density of all possible states (occupied or vacant).

$$D(\Delta E_{\mathrm{R}}) = D_{\mathrm{Fe}^{2+}}(\Delta E_{\mathrm{R}}) + D_{\mathrm{Fe}^{3+}}(\Delta E_{\mathrm{R}}) \qquad \text{(A40)}$$

and if $G(\Delta E_{\mathrm{R}})$ is the probability that the ORP will be reduced, then $1-G(\Delta E_{\mathrm{R}})$ is the probability that the ORP will be oxidized. We deduce that the density of energy states for the reduced state is equal to the product of the density of the energy states $D(\Delta E_{\mathrm{R}})$ by the probability that the state is reduced

$$D_{\mathrm{Fe}^{2+}}(\Delta E_{\mathrm{R}}) = D(\Delta E_{\mathrm{R}}) \cdot G(\Delta E_{\mathrm{R}}) \qquad \text{(A41)}$$

and therefore that

$$D_{\mathrm{Fe}^{3+}}(\Delta E_{\mathrm{R}}) = D(\Delta E_{\mathrm{R}}) \cdot \left(1 - G(\Delta E_{\mathrm{R}})\right) \qquad \text{(A42)}$$

Still taking the ratio of the equations (A41) and (A42), we obtain

$$\frac{G(\Delta E_\mathrm{R})}{1-G(\Delta E_\mathrm{R})} = \frac{D_{\mathrm{Fe}^{2+}}(\Delta E_\mathrm{R})}{D_{\mathrm{Fe}^{3+}}(\Delta E_\mathrm{R})} = \frac{c_{\mathrm{Fe}^{2+}}W_{\mathrm{Fe}^{2+}}(\Delta E_\mathrm{R})}{c_{\mathrm{Fe}^{3+}}W_{\mathrm{Fe}^{3+}}(\Delta E_\mathrm{R})}$$

$$= \frac{c_{\mathrm{Fe}^{2+}}e^{-\frac{\left(\Delta E_\mathrm{R}-\left(\Delta E_\mathrm{R}^\mathrm{o}-\lambda\right)\right)^2}{4kT\lambda}}}{c_{\mathrm{Fe}^{3+}}e^{-\frac{\left(\Delta E_\mathrm{R}-\left(\Delta E_\mathrm{R}^\mathrm{o}+\lambda\right)\right)^2}{4kT\lambda}}} = \frac{c_{\mathrm{Fe}^{2+}}e^{-\frac{\Delta E_\mathrm{R}-\Delta E_\mathrm{R}^\mathrm{o}}{kT}}}{c_{\mathrm{Fe}^{3+}}} = e^{-\frac{\Delta E_\mathrm{R}-\Delta E_{\mathrm{F_R}}}{kT}} \quad \text{(A43)}$$

with

$$\Delta E_{\mathrm{F_R}} = \Delta E_\mathrm{R}^\mathrm{o} + kT\ln\left(\frac{c_{\mathrm{Fe}^{2+}}}{c_{\mathrm{Fe}^{3+}}}\right) \quad \text{(A44)}$$

Thus, the probability $G(\Delta E_\mathrm{R})$for the redox torque to be reduced obtained from the equation (A43) is always of course a Fermi-Dirac distribution at two energy levels

$$G(\Delta E_\mathrm{R}) = \frac{1}{1+e^{\frac{\Delta E_\mathrm{R}-\Delta E_{\mathrm{F_R}}}{kT}}} \quad \text{(A45)}$$

where $\Delta E_{\mathrm{F_R}}$ represents the Fermi energy for of the distribution of the reduced state . This energy represents the reduction energy for which the densities of energy states $D_{\mathrm{Fe}^{2+}}\left(\Delta E_\mathrm{R}\right)$ and $D_{\mathrm{Fe}^{3+}}\left(\Delta E_\mathrm{R}\right)$ are equal. We thus deduce that for this energy we have

$$\frac{c_{\mathrm{Fe}^{3+}}}{c_{\mathrm{Fe}^{2+}}} = e^{-\frac{\Delta E_{\mathrm{F_R}}-\Delta E_\mathrm{R}^\mathrm{o}}{kT}} \quad \text{(A46)}$$

where $\Delta E_{\mathrm{F_R}}$ represents the Fermi energy for of the distribution of the reduced state . This energy represents the reduction energy for which the densities of energy states $D_{\mathrm{Fe}^{2+}}\left(\Delta E_\mathrm{R}\right)$ and $D_{\mathrm{Fe}^{3+}}\left(\Delta E_\mathrm{R}\right)$ are equal. We thus deduce that for this energy we have

$$\tilde{\mu}_{e^-}^\mathrm{S} = \Delta E_{\mathrm{F_R}} \quad \text{(A47)}$$

When the concentrations of Fe^{2+} and Fe^{3+} are equal (standard case), the equation (A47) allows us to define the standard electrochemical potential of the electron in solution

$$\tilde{\mu}_{e^-}^{\ominus,\mathrm{S}} = \mu_{e^-}^{\ominus,\mathrm{S}} - F\chi^\mathrm{S} - F\psi^\mathrm{S} = \Delta E_\mathrm{R}^\mathrm{o} = \Delta E_{\mathrm{F_R}}^{\ominus} \quad \text{(A48)}$$

Thus, in the case of uncharged solutions ($\psi^\mathrm{S} = 0$), the equations (A9) and (A35) allow us to write

$$\alpha_{e^-}^{\ominus,\mathrm{S}} = \alpha_{\mathrm{Fe}^{2+}}^{\ominus,\mathrm{S}} - \alpha_{\mathrm{Fe}^{3+}}^{\ominus,\mathrm{S}} = \Delta E_\mathrm{R}^\mathrm{o} = \Delta E_{\mathrm{F_R}}^{\ominus} \quad \text{(A49)}$$

thus defining the standard real chemical potential of the electron in solution.
